# Rapid rise in atmospheric CO_2_ marked the end of the Late Palaeozoic Ice Age

**DOI:** 10.1038/s41561-024-01610-2

**Published:** 2025-01-06

**Authors:** Hana Jurikova, Claudio Garbelli, Ross Whiteford, Theodore Reeves, Gemma M. Laker, Volker Liebetrau, Marcus Gutjahr, Anton Eisenhauer, Kotryna Savickaite, Melanie J. Leng, Dawid Adam Iurino, Marco Viaretti, Adam Tomašových, Yuchen Zhang, Wen-qian Wang, G. R. Shi, Shu-zhong Shen, James W. B. Rae, Lucia Angiolini

**Affiliations:** 1 School of Earth and Environmental Sciences, University of St Andrews, St Andrews, UK; 2 Department of Earth Sciences, Sapienza Università di Roma, Rome, Italy; 3 GEOMAR Helmholtz-Zentrum für Ozeanforschung Kiel, Kiel, Germany; 4 British Geological Survey, Keyworth, Nottingham, UK; 5 Department of Earth Sciences ‘Ardito Desio’, Università degli Studi di Milano, Milan, Italy; 6 Earth Science Institute, Slovak Academy of Sciences, Bratislava, Slovakia; 7 Nanjing Institute of Geology and Palaeontology, Chinese Academy of Sciences, Nanjing, China; 8 State Key Laboratory for Mineral Deposits Research, School of Earth Sciences and Engineering and Frontiers Science Center for Critical Earth Material Cycling, Nanjing University, Nanjing, China; 9 School of Earth, Atmospheric and Life Sciences, University of Wollongong, Wollongong, New South Wales Australia

**Keywords:** Palaeoclimate, Carbon cycle

## Abstract

Atmospheric CO_2_ is thought to play a fundamental role in Earth’s climate regulation. Yet, for much of Earth’s geological past, atmospheric CO_2_ has been poorly constrained, hindering our understanding of transitions between cool and warm climates. Beginning ~370 million years ago in the Late Devonian and ending ~260 million years ago in the Permian, the Late Palaeozoic Ice Age was the last major glaciation preceding the current Late Cenozoic Ice Age and possibly the most intense glaciation witnessed by complex lifeforms. From the onset of the main phase of the Late Palaeozoic Ice Age in the mid-Mississippian ~330 million years ago, the Earth is thought to have sustained glacial conditions, with continental ice accumulating in high to mid-latitudes. Here we present an 80-million-year-long boron isotope record within a proxy framework for robust quantification of CO_2_. Our record reveals that the main phase of the Late Palaeozoic Ice Age glaciation was maintained by prolonged low CO_2_, unprecedented in Earth’s history. About 294 million years ago, atmospheric CO_2_ rose abruptly (4-fold), releasing the Earth from its penultimate ice age and transforming the Early Permian into a warmer world.

## Abstract (web summary)

A pronounced increase in atmospheric CO_2_ coincided with warming at the end of the Late Palaeozoic Ice Age, according to an 80-million-year-long boron isotope CO_2_ proxy record.

## Main

Earth’s geological history is characterized by transitions between hothouse and icehouse climates, the latter being a relatively less common and ephemeral state^1^. The underlying mechanisms driving these transitions are a matter of still ongoing debate. The canonical view holds that long-term changes in solar radiation or volcanic outgassing are somewhat compensated by the CO_2_ and temperature dependence of silicate weathering, modulating CO_2_ (and other greenhouse gases) to maintain relatively clement climates and habitable conditions^2^. Changes in climate state are thus thought to result from shifts in the balance of these processes, leading to changes in CO_2_. Our knowledge of CO_2_ during much of Earth’s geological history is, however, too limited to fully evaluate CO_2_’s role and governing processes during periods of key climatic transitions^1,3^.

**Fig. 1:**
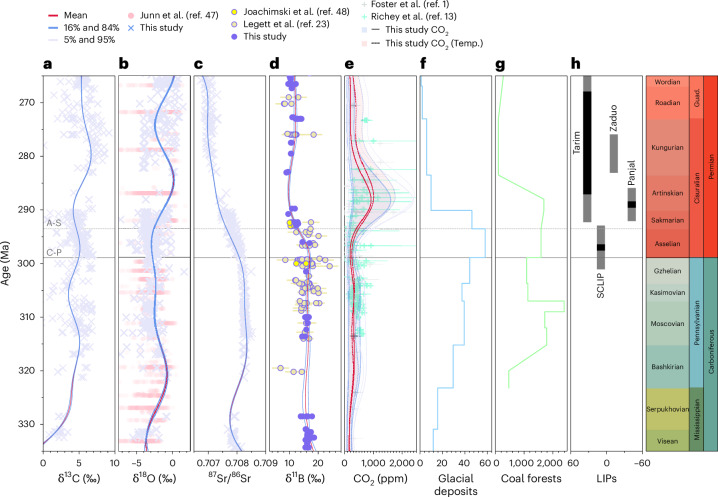
Late Palaeozoic proxy records of climate and seawater chemistry. **a**–**d**, Time-calibrated (GTS 2020) compilation of published and new carbon isotopes (δ^13^C) (**a**), oxygen isotopes (δ^18^O) (**b**), radiogenic strontium isotopes (^87^Sr^/86^Sr) (**c**) and boron isotopes (δ^11^B) (**d**) from preserved brachiopod calcite interpolated with Gaussian process smoothing (showing mean values and 16% and 84% as well as 5% and 95% confidence levels). The error bars show analytical uncertainty (2 s.d.); 0.2‰ in this study and, thus, smaller than symbol size. Previously published carbonate δ^18^O compilation from ref. ^47^ and brachiopod δ^11^B (with 2 s.d.) from refs. ^23,48^ are shown for comparison. **e**, Our boron-derived CO_2_ plotted alongside previously published CO_2_ estimates from soil carbonate- and fossil leaf-based proxies shown with 16% and 84% confidence intervals (ref. ^13^ provides new terrestrial CO_2_ as well as updated values for large part of data compiled in ref. ^1^ for this time interval). The solid line and blue shading show CO_2_ evolution that does not consider the effect of temperature on CO_2_ calculation, and the dashed line and red shading show CO_2_ evolution considering ~9 °C global warming in the Early Permian (‘Temp.’, temperature scenario). **f**, The number of documented glacial deposits (from ref. ^5^). **g**, The extent of tropical coal forests (10^3^ km^2^; from ref. ^30^). **h**, Documented LIPs (following refs. ^38–42^) at their approximate latitudes, with major eruptive phases indicated by black bars. Note that the distribution of glacial deposits and coal forests should be taken as approximate; the paucity of the sedimentary record and the nature of the data make their spatiotemporal distribution and uncertainty difficult to constrain. Stratigraphic column according to International Chronostratigraphic Chart 2023/9 (https://stratigraphy.org/chart). SCLIP, Skagerrak-Centred LIP; Guad., Guadalupian.

The appearance of Gondwanan glaciations^4^ constituting the Late Palaeozoic Ice Age (LPIA) started sometime in the Late Devonian. From about 330 Ma, Earth’s climate gradually cooled through the remainder of the Carboniferous into what has been considered possibly the longest-lived and most extensive and intense of icehouse periods since the radiation of multicellular life^5–7^, with low-latitude sea surface temperatures (SSTs) reaching cooler than modern values, between 19 °C and 24 °C, during much of the LPIA^8–10^. The apex of the LPIA was reached sometime in the latest Carboniferous^11^ to earliest Permian, with major deglaciation achieved by the Artinskian stage of the Early Permian (Fig. 1f). Although glaciogenic deposits have been reported throughout the entire Permian, these appear principally centred over Australia and, thus, are not considered representative of a widespread global glaciation^6,12^. The waxing and waning of Gondwanan ice sheets is thought to have led to eustatic sea level change of ~100 m, translating to about 20 × 10^6^ km^2^ to 37 × 10^6^ km^2^ of ice cover^7^.

Published estimates^1,13^ suggest variable atmospheric CO_2_ during the Carboniferous–Permian, from very low values of <100 ppm up to 2,000 ppm. According to a recent assessment of LPIA CO_2_ (ref. ^13^), CO_2_ concentration dropped from elevated values (~650–1,500 ppm) in the Pennsylvanian to an ~10-million-year-long nadir of ~180–400 ppm spanning the Asselian and Sakmarian stages of the Early Permian. Although the Asselian–Sakmarian CO_2_ nadir seems to broadly coincide with the apex of the glaciation, waning denudation rates of the Variscan orogen, considered responsible for CO_2_ consumption during the Pennsylvanian, would have decreased the CO_2_ sink in the Early Permian, and generally higher (not lower) CO_2_ might be expected based on evidence for increased aridification, demise of wetland tropical forests and volcanism at this time, raising an apparent paradox^13,14^. Reconciling this paradox is central to answering fundamental questions about the icehouse, its demise and the nature of the relationship of the Carboniferous–Permian climate to atmospheric CO_2_ concentrations and requires highly resolved data with a well-calibrated timeline.

Here, we provide an 80-million-year-long time-calibrated boron isotope (δ^11^B)-derived record of atmospheric CO_2_ over the Carboniferous–Permian, accompanied by new strontium (^87^Sr^/86^Sr), carbon and oxygen (δ^13^C, δ^18^O) isotope records and a revised compilation of previously published brachiopod data. We show that CO_2_ remained low throughout the LPIA, with the disappearance of the LPIA being driven by a rapid increase in atmospheric CO_2_ in the Early Permian between 296 million years ago (Ma) and 291 Ma. The long-lasting and stable icehouse conditions of the Late Carboniferous thus ended within a few million years around the Asselian–Sakmarian boundary, replaced by the warmer and drier conditions of the Early Permian. Changing atmospheric CO_2_ therefore played a fundamental role in driving the LPIA dynamics and the Late Palaeozoic climate.

## Calibrated records of the LPIA and its demise

To generate robust records of Late Palaeozoic climate, we assembled a consistent, time-calibrated record of Carboniferous–Permian data from well-preserved brachiopod shells, generating new δ^11^B, ^87^Sr^/86^Sr, δ^13^C and δ^18^O data as well as compiling existing records (Methods). Since fossils are difficult to date directly, we apply an innovative numerical approach to solve numerical ages, employing ^87^Sr^/86^Sr and stratigraphic constrains^15^. We first use ^87^Sr^/86^Sr to assemble a reference ^87^Sr^/86^Sr–time curve representative of the evolution of the marine radiogenic strontium budget across the Carboniferous–Permian using a numerical resampling method. Because Sr is homogeneous in seawater (the ocean mixing time is faster than Sr gain and loss from mid-ocean ridge volcanism and global rivers, 10^3^ years versus 10^6^ years, respectively^16^), coeval samples are statistically more likely to have similar ^87^Sr^/86^Sr than those chronologically farther apart. The ^87^Sr^/86^Sr–time curve is built by assigning a numerical age interval to stratigraphic sections included in the model and where ^87^Sr^/86^Sr data are available. Where radiometric dates are unavailable, numerical ages of stratigraphic intervals are based on biozones. The numerical age of each sample with a known ^87^Sr^/86^Sr is then assigned in a LOESS regression model using random number generator. This approach provides fully comparable and up-to-date brachiopod δ^13^C, δ^18^O, ^87^Sr^/86^Sr and δ^11^B records, calibrated to geologic time scale (GTS) 2020 (see Supplementary Information Section 5 for further details).

Boron isotopes (Fig. 1d), a well-established pH and CO_2_ proxy^17–19^, show stable values throughout much of the Pennsylvanian, terminating 294 ± 1 million years into the Early Permian with an abrupt δ^11^B_brachiopd_ decline of ~8‰. The δ^11^B_brachiopod_ decline indicates a decrease in ocean pH and a corresponding CO_2_ increase in the ocean–atmosphere system, which is unlikely to be explicable by changes in the bulk isotopic composition of boron in seawater (δ^11^B_sw_) given the long residence time of oceanic boron (approximately 10 million years in the modern ocean^20^). The decline in δ^11^B_brachiopod_ coincides with a transitory shift in δ^13^C towards lower values (Fig. 1a) and with the broad decrease in deposition of glacial sediments (Fig. 1f). The drop in δ^11^B_brachiopod_ is superimposed on a gradual long-term decrease in marine ^87^Sr^/86^Sr (Fig. 1c) which lessens from the Sakmarian, following the δ^11^B_brachiopod_ excursion. Combined, our records imply major change in Earth’s ocean–atmosphere system and climate around the Asselian–Sakmarian boundary, linked with a relatively rapid rise in atmospheric CO_2_.

## Carboniferous–Permian CO_2_ and ocean carbonate chemistry

To quantify the magnitude of pH and CO_2_ change, δ^11^B_brachiopod_ data were first adjusted to the δ^11^B of aqueous borate (δ^11^B_4_) by calibrating for biological ‘vital’ effects^21^. The pH was then calculated with the fractionation factor of boron isotopes (*ε*_B_) and by constraining the dissociation constant for boric acid (p*K*_B_^*^) and the δ^11^B_sw_. For robust conversion of δ^11^B_brachiopod_ to δ^11^B_4_ values, we studied the δ^11^B of extant brachiopod taxa living in environments with different pH and devised a new calibration for brachiopod vital effects on the incorporation of boron. p*K*_B_^*^, itself a function of temperature, pressure and seawater composition, was calculated from δ^18^O-derived estimates of Late Palaeozoic SSTs, the depth of the brachiopod habitat and halite fluid inclusion data, respectively. Our methods and calculations are detailed in Supplementary Information, including the limitations based on the key assumptions used in this method, which are discussed further below.

Constraining Carboniferous–Permian δ^11^B_sw_ is not trivial as no direct measurements can be made and there are no existing proxy-based reconstructions at this time. Here, we constrain past δ^11^B_sw_ using a new approach that leverages the common controls shared by the oceanic boron and strontium cycles. As secular changes in both budgets are driven principally by chemical weathering and seafloor spreading, we contend that δ^11^B_sw_ should broadly evolve in a relative fashion similar to that of ^87^Sr^/86^Sr. This similarity is observed within the Cenozoic^22^, but boundary conditions were very different during the Carboniferous–Permian, so we cannot invoke the same straightforward correlation between the two records. Instead, we suggest that the shape of the ^87^Sr^/86^Sr record should loosely inform how δ^11^B_sw_ evolved. This information, coupled to constraints from δ^11^B_4_, allows us to effectively determine plausible δ^11^B_sw_ and, therefore, plausible pH evolutions (Supplementary Information Section 6). We first attempt to minimize the amount of pH change we reconstruct by maximizing the change in δ^11^B_4_ explained by δ^11^B_sw_—testing against the null hypothesis that our record represents no pH change. The rapidity of the change in δ^11^B_4_ around 294 Ma is incompatible with δ^11^B_sw_ as a driver; thus, we find a robust change in ocean pH at this time.

**Fig. 2:**
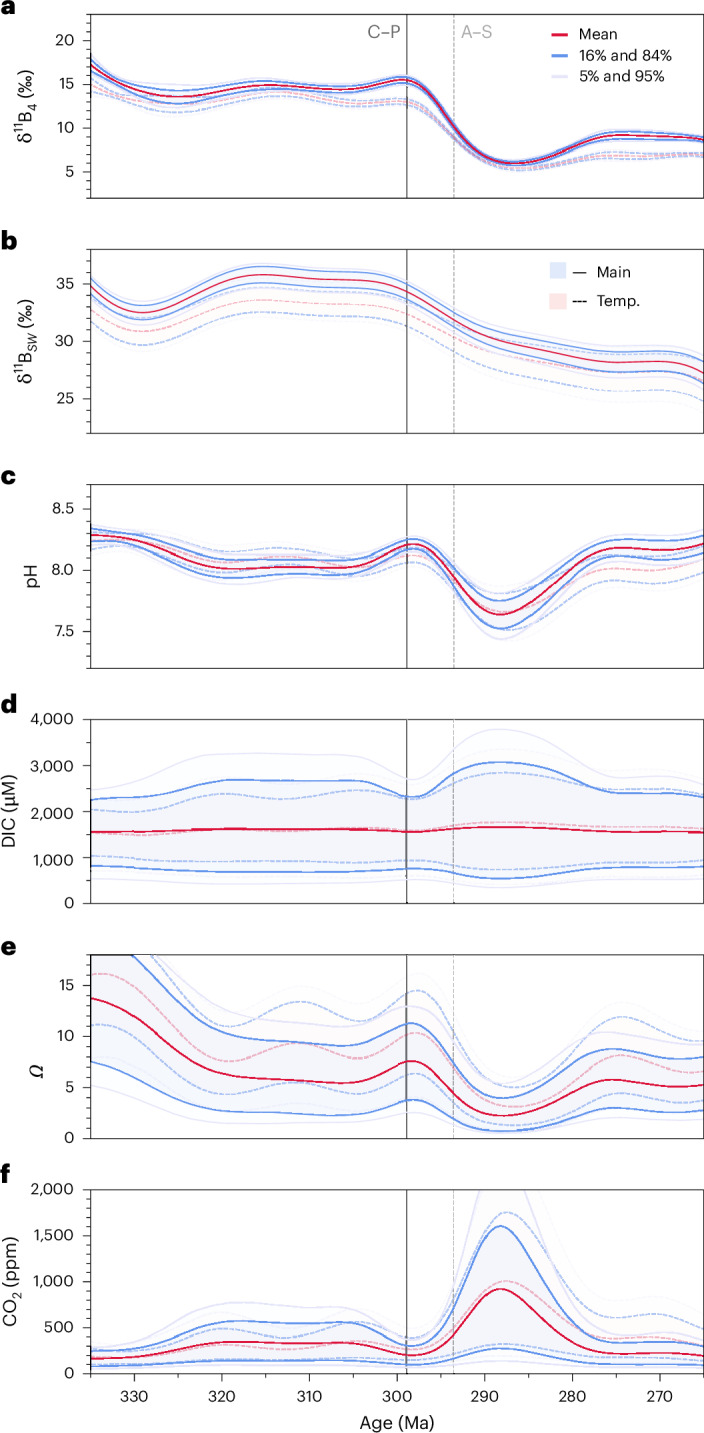
Carboniferous–Permian evolution of boron and carbon system chemistry. **a**, The boron isotope composition of aqueous borate (δ^11^B_4_ from δ^11^B_brachiopod_). **b**–**f**, The boron isotope composition of seawater (δ^11^B_sw_) (**b**), ocean pH (**c**), DIC concentration (**d**), ocean saturation state (*Ω*) (**e**) and atmospheric CO_2_ (**f**); reconstructed using our main scenario (Main), and a scenario considering an ~9 °C warming in the Early Permian (‘Temp.’, temperature scenario). Each panel shows mean values and 16% and 84% as well as 5% and 95% confidence levels. C–P, Carboniferous–Permian boundary; A–S, Asselian–Sakmarian boundary.

The background climate and carbonate system state during the Carboniferous affects the magnitude of the perturbation we reconstruct. The nature of the δ^11^B–pH relationship is such that the higher the background seawater pH, the smaller the ∆pH for a given δ^11^B excursion. However, background pH cannot be too high, else we would reconstruct unreasonably low atmospheric CO_2_ concentration and/or impossibly high saturation state (*Ω*), in addition to the potential direct impact such conditions would have on marine organisms. Our algorithm uses Markov Chain Monte Carlo to balance these factors and to find a space within which all parameters are viable within broad, conservative ranges (see Methods and the paragraph below). This approach produces a background climate state during the LPIA with pH of ~8.0, CO_2_ of ~300 ppm, dissolved inorganic carbon (DIC) of about 1,500 μmol kg^−1^ and *Ω* of around 6 (Fig. 2).

Temperature also affects the magnitude of atmospheric CO_2_ reconstructed. Given the lack of information on the evolution of the oxygen isotope composition of seawater (δ^18^O_sw_) during the Late Palaeozoic, SST reconstruction from δ^18^O relies on assumptions being made about δ^18^O_sw_. The most conservative approach that can be taken is that of constant δ^18^O_sw_ applied here (main scenario). This assumption may, if anything, result in an underestimation of Permian atmospheric CO_2_ due to decreased solubility of CO_2_ at warmer temperatures. To show the effect of warming on our CO_2_ calculations, we also provide a second reconstruction that considers an SST increase of ~9 °C from Late Carboniferous to Early Permian^8^ (Figs. 1e and 2f). Both scenarios produce comparable CO_2_ trends, with the later suggesting on average ~200 ppm higher CO_2_ following the Sakmarian.

Around the Asselian–Sakmarian boundary, δ^11^B and pH decrease relatively rapidly, but establishing the impact of this pH change on CO_2_ is complicated by the need for a second carbonate system parameter. We overcome this difficulty by first establishing a null hypothesis—that atmospheric CO_2_ did not change—then calculating how our secondary carbonate system parameters (that is, besides pH) would have had to vary to cause this to occur. To do this, we take a hypothetical DIC evolution and apply a multiplier to it, allowing the impact of changing pH on CO_2_ to be dampened, amplified or completely inverted by changing DIC. The feasibility of the chosen DIC evolution is validated by ensuring both atmospheric CO_2_ and *Ω* stay within reasonable limits. This method demonstrates that it is very unlikely that the pH change at ~294 Ma can indicate anything other than a sizeable increase in atmospheric CO_2_ (Supplementary Information Section 6).

**Fig. 3:**
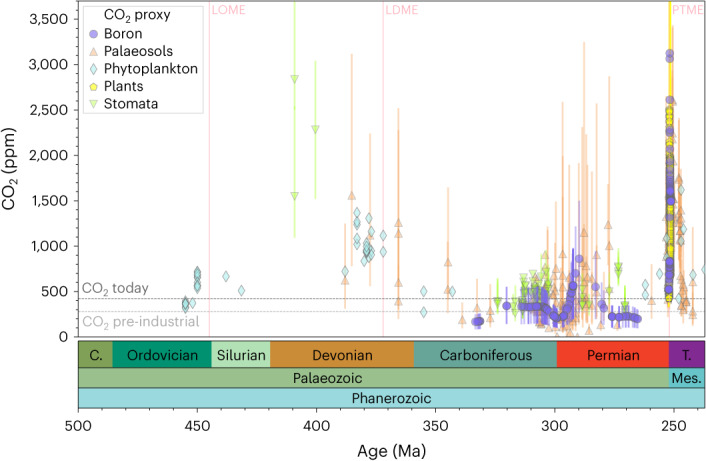
Palaeozoic CO_2_ from different proxies. Boron: this study and ref. ^17^; palaeosols: refs. ^1,13,25,49^; phytoplankton: ref. ^50^; plants: ref. ^51^; stomata: refs. ^1,13,25^ (and references therein). The error bars show 16% and 84% confidence intervals. LOME, Late Ordovician Mass Extinction; LDME, Late Devonian Mass Extinction; PTME, Permian–Triassic Mass Extinction. C., Cambrian; T., Triassic; Mes., Mesozoic.

The only alternate explanation is to invoke a substantial decrease in DIC concentration coeval with pH fall. We believe this to be unlikely, as any DIC decrease larger than that shown in Fig. 3 would result in average *Ω* < 1 and, mechanistically, ocean acidification of this kind is typically associated with CO_2_ injection into the atmosphere–ocean system, which would be expected to increase DIC. Overall, even though we have taken care to allow the possibility of invariant pH and/or CO_2_ over this time, our conservative approach suggests a major shift in ocean pH, *Ω* and atmospheric CO_2_ concentrations around the Asselian–Sakmarian boundary.

Following the elevated CO_2_ event around the Asselian–Sakmarian boundary, interestingly, our CO_2_ system solutions favour a gradual return to higher pH and lower CO_2_ values again in the Kungurian, despite δ^11^B_4_ remaining low. Unlike the elevated CO_2_ event, this gradual rebound is influenced by δ^11^B_sw_, which shows a decreasing trend throughout the Permian. Future δ^11^B_sw_ constraints will help refine the magnitude of this change and the resulting pH and CO_2_ values. We note, however, that there is geological and proxy evidence for cool conditions in the Early–Middle Permian^6,8,12^, and both increased chemical weathering and seafloor spreading rates at this time would favour a decrease in δ^11^B_sw_, as also suggested from seawater boron isotope budget modelling^23^, supporting our independent model.

## Rapid release from an intense icehouse by LIP CO_2_ emissions

Available estimates indicate that, throughout Earth’s history, atmospheric CO_2_ has generally remained above Pleistocene glacial levels of ~180 ppm (refs. ^1,13,19,24^). Our reconstruction shows sustained low CO_2_ of about 330 ± 210 ppm during much of the LPIA, which declines during its apex, reaching minimum values of ~200 ± 100 ppm (84% confidence levels; Fig. 3) at ~298 Ma, before increasing rapidly from ~294 Ma. The boron-derived CO_2_ fits well with the broad view of Late Palaeozoic CO_2_ inferred from soil carbonate and stomata^6,13,25^ (Fig. 3); we further refine the resolution, timing—including early occurrence of the CO_2_ nadir around the Carboniferous–Permian boundary—and magnitude of Late Palaeozoic CO_2_ change and, notably, reveal the rising CO_2_ starting from around the Asselian–Sakmarian boundary.

Our CO_2_ record extends the prevalence of low glacial CO_2_ to the Visean stage, which, exacerbated by ~3% lower solar luminosity, would have caused icehouse conditions. To maintain low CO_2_, CO_2_ outgassing from the solid Earth must have been either reduced or effectively consumed. The role and relative contribution of these processes to the glaciation is, however, difficult to disentangle from available proxy evidence. High-enough CO_2_ consumption to sustain such low atmospheric CO_2_ has been explained by enhanced chemical weathering, as collision between Laurasia and Gondwana in the Carboniferous led to the uplift of the Greater Variscan (Hercynian) mountain plateau^26^. The Variscan orogenic belt is thought to have been of wide meridional extent as it drifted northwards into the equatorial humid belt by the Pennsylvanian, where the exposure of calcium- and magnesium-rich Variscan crystalline rocks could have facilitated enhanced silicate weathering^27,28^. Although Palaeozoic seafloor spreading rates (and resultant CO_2_ outgassing) are challenging to constrain due to subduction of oceanic crust into the mantle, subduction flux reconstructions from full-plate models suggest sustained decline in outgassing rates throughout the LPIA^29^, providing a plausible alternative mechanism. Diminished radiative forcing from sulfate aerosols may have also contributed to the peak glaciation^5^, warranting further research.

Weathering is thought to have decreased in intensity by the Early–Middle Permian. With steady drifting of Pangea into the northern hemisphere and aridification in response to growing continentality during Pangea’s transformation, available land mass in the intense weathering zones of low latitudes was reduced, as was the arial extent of reactive land surfaces available to weathering^27,29^. Some CO_2_ would have also been drawn down in organic carbon as tropical coal forests expanded on land, although they are unlikely to be the main cause of the CO_2_ drawdown, as major changes in their spatial extent pre- and post-date the LPIA (Fig. 1g; ref. ^30^). Owing to their deep-rooting systems, vascular plants may have also in part contributed to enhancing silicate weathering^31^. The described weathering patterns are well reflected in the enhanced marine ^87^Sr^/86^Sr (Fig. 1c) signalling flux of continental crust-derived radiogenic Sr to the ocean during much of the Pennsylvanian and its posterior decrease, although increased spreading due to the opening of the Neotethys could also equally well explain the observed Cisuralian (Early Permian) ^87^Sr^/86^Sr decline^32,33^ and is supported by subduction flux reconstructions^29^.

**Fig. 4:**
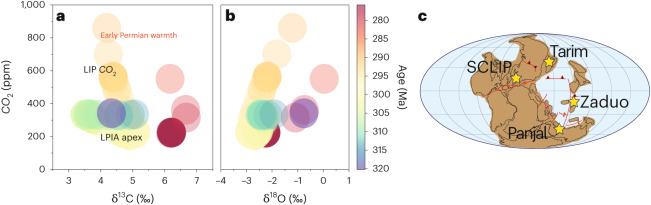
Climate states of the Late Palaeozoic. **a**,**b**, Paired brachiopod carbon isotopes (**a**) and oxygen isotopes (**b**) versus atmospheric CO_2_. The transition from Carboniferous LPIA to Early Permian warmhouse was driven by a rapid CO_2_ release around the Asselian–Sakmarian boundary (about 294 ± 1 Ma), which leads to a modified trajectory in δ^13^C-CO_2_ (**a**) and δ^18^O-CO_2_ (**b**) space. The CO_2_ release event coincides with the emplacement of Skagerrak-Centred LIP (SCLIP); the location of other Early Permian LIPs (Tarim, Panjal and Zaduo) that have been hypothesized to have influenced Permian climate is illustrated for comparison. Palaeogeographic map following ref. ^27^.

The CO_2_ rise, however, occurred much more rapidly than the gradually decreasing strength of silicate weathering or increased spreading, reflected by the gradually changing seawater ^87^Sr^/86^Sr and δ^11^B_sw_, could have facilitated. Previous seawater reconstruction also showed that changes around the Asselian–Sakmarian boundary cannot be explained by δ^11^B_sw_ (ref. ^23^). The increasing CO_2_ coincident with moderately decreasing δ^13^C is most convincingly explained by mantle-sourced carbon degassing^34^ characteristic of emplacement of a large igneous province (LIP; Fig. 4). Rapid warming at this time is also supported by stratigraphic evidence for global sea level rise from the base of the Sakmarian^35–37^.

**Fig. 5:**
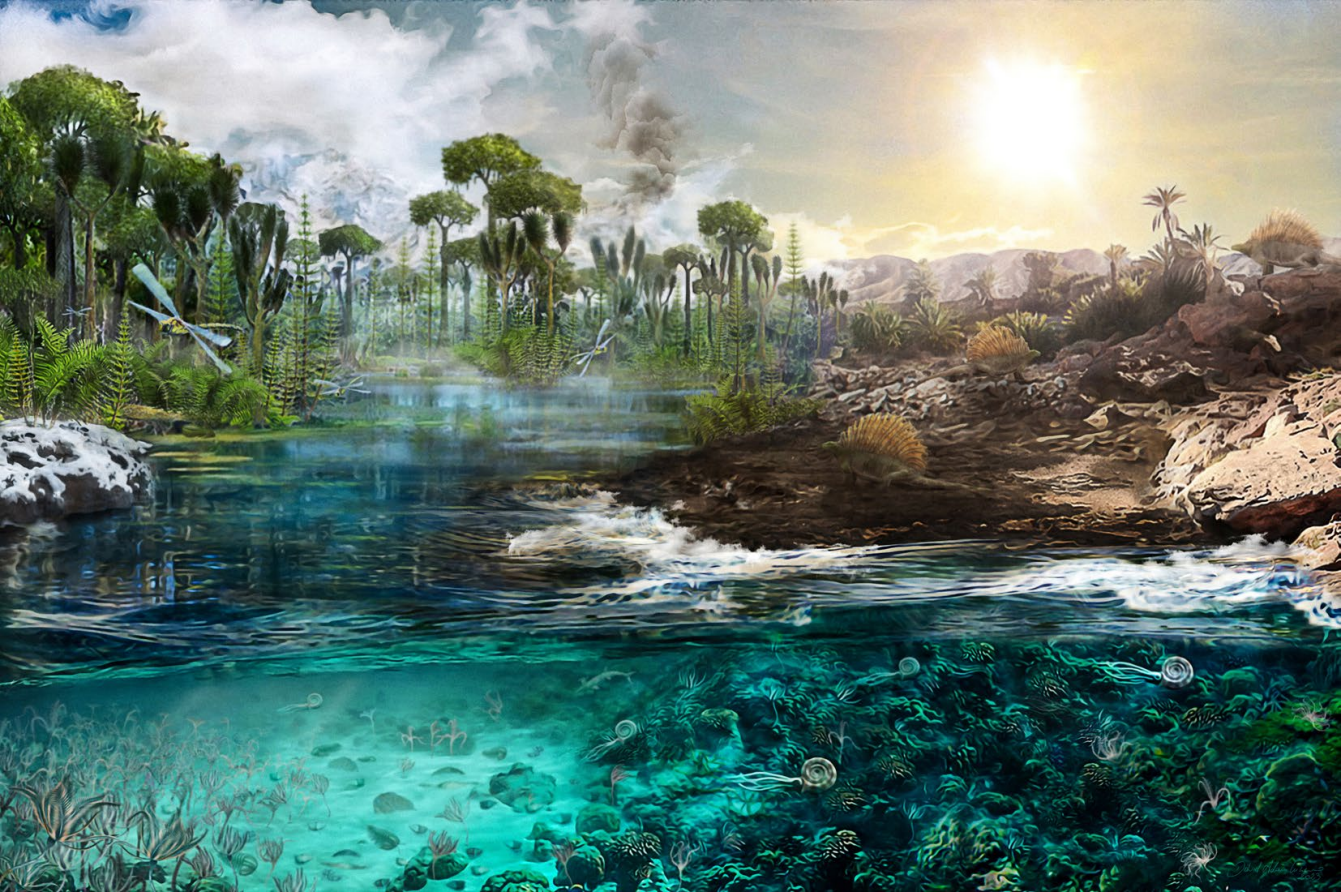
The end of the LPIA and the dawn of the Early Permian warmth. A relatively rapid rise in atmospheric CO_2_ approximately 294 Ma released the Earth from its penultimate icehouse (left) and transitioned the world to a warmer and drier climate of the Early Permian (right). Palaeo-artistic rendering based on findings of this study and previously published literature (Supplementary Information Section 7).

The eruption of at least four different LIPs in the Early–Middle Permian (that is, the Skagerrak-Centred, the Panjal, the Zaduo and the Tarim; Fig. 1h) has been hypothesized to have played some role in the Carboniferous–Permian climatic transitions^38–40^, although their link has thus far not been comprehensively demonstrated. The onset of the rise in CO_2_ reconstructed here coincides most closely with the eruption of the Skagerrak-Centred LIP dated 297 ± 4 Ma, which has been suggested to be capable of releasing voluminous quantities of CO_2_ during magmatic degassing, due to rapidly spreading flood basalts covering vast areas >0.5 million km^2^ (ref. ^41^). Maximum CO_2_ coincides with the main magmatic phase of the Tarim LIP and the Panjal LIP, which might have also contributed to maintaining elevated CO_2_ levels. We make the case that, similar to many other episodes of major environmental change in Earth’s history^42,43^, the end of the LPIA was driven by LIP CO_2_ emissions (Fig. 4); however, rather than a mass extinction, it seems to have led to a biodiversity increase^44^. The elevated CO_2_ established a period of transient Early Permian warmth, which profoundly transformed the environment, ecology and evolution of global fauna and flora, setting the stage for the rise of amniotes (that is, reptiles^45^; Fig. 5). Our data thus provide further evidence that carbon release from large-scale magmatism was a leading cause of transitions in climate and environments in the geological record.

The gradual onset, nature and even duration of the LPIA appear to share some interesting parallels with the current Late Cenozoic Ice Age (LCIE), which started at ~34 Ma with the glaciation of Antarctica. Similarly to the LPIA, the cause of the LCIE has been linked to a major orogen in low latitudes (Eocene–Oligocene uplift and faulting of the Tibetan Plateau) and a reduction in seafloor spreading driving long-term decline in CO_2_ (refs. ^28,46^). It remains to be seen whether a future LIP event will similarly end the current LCIE, or whether the emergence of an evolutionarily extremely successful organism (that is, humans) may lead to a geological-scale climate transition.

## Methods

A collection of prescreened well-preserved ‘silky’ brachiopod shells from Iran, Oman, China, Australia and the United Kingdom spanning the Visean (Carboniferous) through to Wordian (Permian) stages was analysed for boron (δ^11^B), strontium (^87^Sr/^86^Sr), carbon (δ^13^C) and oxygen (δ^18^O) isotopes, and Element/Ca composition (Li, B, Na, Mg, Al, Mn, Fe, Sr, Ba, Nd and U). Boron isotope and elemental analyses were carried out at the University of St Andrews, United Kingdom, and GEOMAR Helmholtz Centre for Ocean Research Kiel, Germany, on a Thermo Scientific Neptune Plus MC-ICP-MS (multicollector inductively coupled plasma mass spectrometer) and either Agilent 8900 QQQ-ICP-MS (triple quadrupole) or Agilent 7500x Q-ICP-MS (quadrupole inductively coupled plasma mass spectrometer) following protocols previously detailed in refs. ^17,18,21,52,53^. The analytical reproducibility, assessed by repeat measurements of standards processed and purified alongside samples, was <0.2 ‰ (2 s.d.). Strontium isotope ratios were measured at GEOMAR, Kiel using a Thermo Scientific Triton TIMS (thermal ionization mass spectrometer) with analytical precision <0.000002 (2 s.d.). Carbon and oxygen analyses were performed at the British Geological Survey in Keyworth, United Kingdom, on an Isoprime PreciSION dual inlet mass spectrometer with Multiprep device. The analytical reproducibility was better than 0.09‰ for δ^18^O and 0.03‰ for δ^13^C (1 s.d.).

The generated data alongside previously published brachiopod values were assembled into a Carboniferous–Permian δ^11^B, ^87^Sr/^86^Sr, δ^13^C and δ^18^O compilation (Supplementary Data 1). Numerical ages were generated using a LOESS ^87^Sr/^86^Sr–time model, anchored in radiometric dates and biostratigraphy calibrated to GTS 2020. Measured δ^11^B_brachiopod_ values were converted to δ^11^B_4_ (δ^11^B of borate ion) using a vital effects calibration based on modern brachiopods. The data compilation was assimilated into a Bayesian framework for interpolation and to quantify the pH and CO_2_ system. We used a Gaussian process^22^ to interpolate the data into records, and a Markov Chain Monte Carlo approach to integrate the geochemical records with ancillary constraints required for the calculation of pH and CO_2_ from δ^11^B values (that is, temperature, pressure, seawater major ion composition and δ^11^B_sw_). δ^11^B_sw_ was constrained by defining a plausible value range from the δ^11^B_4_–pH relationship, with its evolution guided by the ^87^Sr/^86^Sr record. The reported results (posterior metrics) are based on 10,000 iterations of the Bayesian framework. For further details on materials and methods, we refer the reader to Supplementary Information.

## Online content

Any methods, additional references, Nature Portfolio reporting summaries, source data, extended data, supplementary information, acknowledgements, peer review information; details of author contributions and competing interests; and statements of data and code availability are available at 10.1038/s41561-024-01610-2.

## Supplementary information

Supplementary Information Supplementary Sections 1–7, Figs. 1–14 and references.

Supplementary Data 1 Carboniferous–Permian δ^11^B, ^87^Sr^/86^Sr, δ^13^C and δ^18^O data compilation.

Supplementary Data 2 Interpolated records (including pH and CO_2_) paired to δ^11^B samples, main scenario.

Supplementary Data 3 Interpolated records (including pH and CO_2_) at 100 kyr resolution, main scenario.

Supplementary Data 4 Interpolated records (including pH and CO_2_) at 100 kyr resolution, second (temperature) scenario.

## Data Availability

All data from this study are available in Supplementary Data 1–4 and via Zenodo at 10.5281/zenodo.14040601 (ref. ^54^).

## Code availability

Code from this study is available on GitHub at https://github.com/St-Andrews-Isotope-Geochemistry/Carboniferous-Permian-d11B.
