# Supplementary material for: Rapid rise in atmospheric CO2 marked the end of the Late Palaeozoic Ice Age
Source: Nat Geosci. 2025 Jan 6;18(1):91–7. doi: 10.1038/s41561-024-01610-2 (PMC11732749; doi:10.1038/s41561-024-01610-2)
Supplement: Supplementary file 1 — Supplementary Sections 1–7, Figs. 1–14 and references. [file 41561_2024_1610_MOESM1_ESM.pdf]

nature geoscience

Supplementary information

https://doi.org/10.1038/s41561-024-01610-2

Rapid rise in atmospheric CO_2 marked the end of the Late Palaeozoic Ice Age

In the format provided by the authors and unedited

Jurikova et al.

Supplementary Information

Contents:

1. Stratigraphy and samples

2. Sample preparation and analysis

3. Preservation and diagenetic screening

4. Vital effects and a new $\delta^{11}B$ calibration for brachiopods

5. $^{87}Sr/^{86}Sr$-time curve modelling

6. Modelling of $\delta^{11}B_{sw}$, ocean pH and atmospheric CO_2

7. Palaeo-artistic reconstruction of the Late Palaeozoic world

References

Accompanying Supplementary Data:

Data S1. Carboniferous-Permian $\delta^{11}B$, $^{87}Sr/^{86}Sr$, $\delta^{13}C$ and $\delta^{18}O$ data compilation.

Data S2. Interpolated records (including pH, CO_2) paired to $\delta^{11}B$ samples, main scenario.

Data S3. Interpolated records (including pH, CO_2) at 100 kyr resolution, main scenario.

Data S4. Interpolated records (including pH, CO_2) at 100 kyr resolution, second scenario.

Also available on Zenodo: https://doi.org/10.5281/zenodo.14040601

Accompanying Code:

https://github.com/St-Andrews-Isotope-Geochemistry/Carboniferous-Permian-d11B

1. Stratigraphy and samples

Our record is based on new brachiopod data measured in this study and a compilation of previously published data from the literature (Data S1). We compiled, to the best of our knowledge, all available high-fidelity brachiopod $\delta^{13}C$, $\delta^{18}O$, $^{87}Sr/^{86}Sr$, and $\delta^{11}B$ datasets spanning the Carboniferous-Permian from ~335 to ~265 Ma to construct 80-million-year long records of climate and seawater chemistry. The data compiled comes from: Angiolini et al. (2006, 2007, 2008, 2012, 2019), Garbelli et al. (2016, 2019, 2022a); Grossman et al. (1991, 1993, 2008); Joachimski et al. (2005); Korte et al. (2005, 2006), Legett et al. (2020), Mii et al. (1997), Morante (1999), Popp et al. (1986), Veizer & Composton (1974), Veizer et al. (1999), Vollstaedtet al. (2014) and Wang et al. (2020; 2021).

New key measurements were made on brachiopod collections consisting of samples from Iran (Bahrammanesh et al., 2011, Brenckle et al., 2009, Berra et al., 2017), UK (Angiolini et al. 2012; 2019; Nolan et al. 2017), Oman (Angiolini et al. 2006; 2007; 2008; Viaretti et al. 2022), China (Wang et al., 2020; 2021), and Australia spanning the Lower Carboniferous to the Middle Permian (see Table S1 for an overview; all geochemical data is available in Data S1).

Horizon	Locality	Formation	Country	Age
IR	Alborz Mts.	Mobarak Fm.	North Iran	Tournaisian and early Visean
AJ	Jandaq	Godar-e-Siah Complex	Central Iran	Moscovian
RCK	Ricklow	Eyam Lmst. Fm.	UK	late Visean
OAW	Once a Week	Eyam Lmst. Fm.	UK	late Visean
IBL	Ible	Monsal Dale Lmst. Fm.	UK	late Visean
	Fife	Lower Lmst. Fm.	UK	late Visean
WKJ	Wadi Khawr al Jaramah	Qarari Unit	Oman	late Kungurian-early Roadian
JX3	Jebel Qarari	Qarari Unit	Oman	late Kungurian-early Roadian
OL, OM	Haushi	Saiwan Fm.	Oman	Sakmarian
XK	Xikou	Xikou, Shuixiakou	China	Moscovian and Asselian
AU	Sydney Basin		Australia	Sakmarian-Wordian

Table S1. *List of formations from which our brachiopod samples came from. Further details are available in the accompanying brachiopod compilation database.*

The specimens from Iran come from different fossil localities of North and Central Iran.
The oldest specimens are Tournaisian and they have been collected in marlstones and bioclastic limestones of the middle part of the Mobarak Fm. at the Abrendan section (36°2147.6"N, 54°18'59.9"E), Alborz Mountains, North Iran (Bahrammanesh et al. 2011). The depth of the depositional setting was below the normal wave base, with substrate varying from muddy to hard. The lower Visean specimens have been collected from the upper part of the Mobarak Fm. at the Abnak section (North Iran), in an interval of shale and marlstone intercalations in the shallow water oolitic and bioclastic limestones which characterize the upper part of the formation (Brenckle et al. 2009). This indicates deposition in a more protected and quieter environment when compared with the high-energy setting of the upper part of the Mobarak Fm. The estimated palaeolatitude for both localities is 45–50°S (Bahrammanesh et al. 2011). The Moscovian specimens from the Godar-e-Siah Complex of Jandaq, Central Iran come from sandy limestones of the Godar-e-Siah northern and central outcrops (33.91°N, 54.26°E; 33.951°N, 54.263°E) deposited in a shallow water, tidally- influenced environment (Berra et al. 2017). The estimated palaeolatitude is about 10°–15°N.

The specimens from Oman come from outcrops of the Saiwan Fm. in the Haushi area (20°52'04"N, 57°36'27"E–20°52'26"N, 57°36'26"E) in central interior Oman, and are mid to late Sakmarian in age (Angiolini et al., 2006, 2007, 2008). The Saiwan Fm. consists of cross-

laminated bioclastic sandstones, mudrocks, and sandy calcarenites passing upward to coarse-grained and cross-laminated sandy calcarenites, bioclastic limestones, and marlstones, deposited around or just below the fair-weather wave base at a palaeolatitude of 40°–45°S (Angiolini et al., 2003). At the base, the Saiwan Fm. is characterized by the occurrence of the *Pachycyrtella* bed interpreted by Angiolini et al. (2003) and Angiolini (2007) as recording a physically-controlled pioneer palaeocommunity colonizing high-nutrient and high-energy settings along the Neotethys margin at the end of the Late Palaeozoic Ice Age (LPIA). Angiolini (2007) initially considered the large biovolume of the thick-shelled *Pachycyrtella omanensis* as an adaptation to cool climate conditions. However, as suggested by Angiolini et al. (2003) and Shiino & Angiolini (2014), the large biovolume of *P. omanensis* is best attributed to a marked increase in the effective surface area of the lophophore enabling fast growth rates. Their thick shells, high growth rates and increased weight of the ventral umbo enhance physical stability and represent adaptations to high-energy flow regimes, without altering the shell morphofunction to generate passive feeding flows (Shiino & Angiolini 2014). The shell of *P. omanensis* is mostly made of secondary fibrous layer with no columnar tertiary layer, suggestive of low carbonate saturation state of seawater (Ω), as described by Ye et al. (2018) based on recent brachiopods from low carbonate saturation settings. For instance, the recent Antarctic *Liothyrella uva* living in waters with Ω ~2 does not form a tertiary layer, while *L. neozelanica* with a tertiary layer is commonly found in environments with Ω ~4. The onset of the decline in Ω inferred from *P. omanensis* is in line with our boron isotope- and model-based evidence of rapid decline in ocean pH and Ω as a result of rapid CO_2 increase following the Asselian-Sakmarian boundary.

Other specimens from Oman have been collected from the Wadi Khawr al Jaramah outcrops (22°27'38"N, 59°40'51"E) of the allochthonous Qarari Unit west of Ras al Hadd; one specimen (JX) come from a hill just north of Jebel Qarari (21°55'07"N, 59°27'42"E). These scattered outcrops of marly bioclastic limestones are richly fossiliferous and have been given a late Kungurian-early Roadian age, so they straddle the Cisuralian-Guadalupian boundary (Viaretti et al. 2022). They have been interpreted as deposited at several tens of metres of depth around or below the storm wave base at a palaeolatitude of about 30°S (Garbelli et al. 2022b).

Specimens from the UK come from the *Gigantoproductus* beds of the Monsal Dale Limestone Fm. at Ible (53.111N, 1.630W; Angiolini et al. 2012) and of the Eyam Limestone Fm. at Ricklow Quarry (53.192N, 1.755W) and Once a Week Quarry (53.209N, 1.766W; Nolan et al. 2017; Angiolini et al. 2019), both late Visean (Brigantian) in age. Several specimens were also collected from the Lower Limestone Group from the East Neuk of Fife (Scotland; Fielding et al. 1988).

The Monsal Dale Limestone Fm. consists of massive, cyclic, bioturbated bioclastic limestones, with floatstone beds with gigantoproductins and mud mounds in its upper part. The Eyam Limestone Fm. consists of fossiliferous limestones which are very rich in gigantoproductins, corals, crinoids, and dark mudstone intercalations; mud mounds occur in the middle and in the upper part of the formation (Carniti et al., 2023). The two formations are separated by an unconformity recording regional subaerial exposure and both have been deposited in settings varying from above to below the normal wave base at equatorial palaeolatitudes (4°S). The shell growth and geochemistry of the shells of the species of *Gigantoproductus* have been investigated in detail by Angiolini et al. (2012, 2019) who showed that their gigantic size and thick shell mostly made of a columnar tertiary layer was due to a mixotroph lifestyle in shallow water mobile substrates, deriving energy and nutrients both from photosymbiotic microbes and from filtered particulate food. One interesting feature of the shell of the species of

Gigantoproductus is that they record a seasonal temperature variation which is much higher than what expected at comparable equatorial latitudes; Angiolini et al. (2012) reported $\delta^{18}O$ variations of about 1‰, whereas low latitude brachiopods from the Upper Permian of the Dolomites show a variation of 0.4 ‰ (Garbelli et al. 2022a), the same shown by modern *Tridacna gigas* shells from South China (Ma et al. 2020). This means that the seasonal change, whether caused by temperature or by moonsonal precipitation, was almost doubled at the palaeoequator in the late Visean. As seasonality increases during cold climates and intensification of glacial events, we can infer that the upper Visean brachiopod archive recorded high seasonality as a far-field proxy of the onset of sustained Gondwanan glaciation in the late Visean (Montañez, 2022).

Specimens from China are collected from the Xikou section (33.23° N, 109.38°E), at Zhen'an County in Shaanxi Province. The Early Permian carbonate sequences were divided into four different formations by Ding et al. (1989) mainly according to the fusulinid. Lithologically, the Sanlichong to Wulipo formations consisted of thickly-bedded bioclastic limestones with abundant fusulinids and were assigned to the Cisuralian to lower Guadalupian stages. The occurrence of *Pseudoshwagerina* defines the boundary between the Carboniferous and the Permian periods. The occurrences of *Pseudofusulina, Pamirina, Brevaxina, Neoschwagerina*, and *Presumatrina* define the base of the Sakmarian, Artinskian, Kungurian, Roadian, and Wordian stages, respectively (Wang et al., 2021).

2. Sample preparation and analyses

Samples were prepared and measured at the St Andrews Isotope Geochemistry (STAiG) laboratory of the University of St Andrews, UK and at GEOMAR Helmholtz Centre for Ocean Research Kiel, Germany following previously detailed protocols (Jurikova et al., 2019; 2020a; 2020b). In brief, approximately 2–4 mg of pristine ' silky' brachiopod calcite was collected from each specimen by avoiding the primary layer and only sampling the secondary, and when present, the tertiary layer. Samples were split into two parts – one part was measured for $^{87}Sr/^{86}Sr$ and the other for Element/Ca and $\delta^{11}B$. The latter was treated with clay removal (using ultrasonication in boron-free ultra-pure water Milli-Q, 18.2 Ω) and oxidative cleaning steps (using 1% H_2O_2 buffered with 0.1 M NH_4OH at 80 °C) prior to dissolution. Clean samples were dissolved with the aid of ultrasonication in 0.5 M HNO_3. A small aliquot of dissolved sample (~10 % volume) was first used to determine Element/Ca composition on an Agilent 7500x Q-ICP-MS or Agilent 8900 QQQ-ICP-MS. The remainder of the dissolved sample underwent boron purification from the $CaCO_3$ matrix using boron-specific anion exchange resin, Amberlite IRA 743, crushed and sieved to 63–106 μm (Trudgill et al., 2024). Brachiopod samples were processed alongside known standards and total procedural blanks (TPBs), including certified reference materials JCp-1 (recent *Porites sp.* coral powder; Gutjahr et al., 2020) and RM NIST 8301 Foram (synthetic foraminifer solution; Stewart et al., 2021) and an in-house brachiopod standard MVS-1 (recent *Magellania venosa* powder; Jurikova et al., 2019). Purified boron solutions were measured on a Thermo Scientific Neptune Plus MC-ICP-MS, as described in Xu et al. (2024). Instrumental mass bias was corrected by standard sample-bracketing with a NIST SRM 951 solution. The average $\delta^{11}B$ for ERM-AE121 measured in sequences alongside samples was 19.53±0.24 ‰ (2SD, n = 6) at GEOMAR, Kiel and 19.65± 0.14 ‰ (2SD, n = 9) at the University of St Andrews. JCp-1 was 24.21±0.19 ‰ (2SD, n = 4) at GEOMAR Kiel and 24.22±0.08 ‰ (2SD, n = 4) at the University of St Andrews. All TPBs were <0.1% of the typical sample size and hence negligible.

Strontium isotope analyses were carried out at GEOMAR Helmholtz Centre for Ocean Research Kiel, Germany, following previously described protocols (e.g. Vollstaedt et al., 2014). Sr was extracted using Sr-spec ion chromatography and measured using a Thermo Scientific TRITON TIMS. The analytical reproducibility was <0.000002 (2SD). All generated $^{87}Sr/^{86}Sr$ were normalized to the accepted NIST SRM 987 $^{87}Sr/^{86}Sr$ values of 0.710248.

Carbon and oxygen isotope analyses were performed at the British Geological Survey, Keyworth, UK. Approximately ~100 μg of brachiopod shell calcite was dissolved in 100% H_3PO_4, with the oxygen and carbon isotope ratios ($^{13}C/^{12}C$ and $^{18}O/^{16}O$) measured using an Isoprime PreciSION dual inlet mass spectrometer plus Multiprep device. $\delta^{13}C$ and $\delta^{18}O$ are reported as ‰ deviations of the isotope ratios calculated to the V-PDB scale using a within-run laboratory standard Keyworth Carrera Marble (KCM) calibrated against the international standards (NBS18 and NBS19). Analytical reproducibility was better than 0.09‰ for $\delta^{18}O$ and 0.03‰ for $\delta^{13}C$ (1SD).

3. Shell preservation and diagenetic screening

Preservation of brachiopod shells was assessed using diagenetic screening based on microstructural analyses under a scanning electron microscope (SEM; using a JEOL JSM-IT500 LA) and cathodoluminescence analyses (CL; using a RELIOTRON system connected to a Nikon Eclipse 100LV microscope at the University of Milano-Bicocca). Diagenetic screening of Visean brachiopod shells from UK was available from published literature, including some EBSD analyses of species of *Gigantoproductus* (Angiolini et al., 2019), which lead to consider the specimens OAW203 and RCK221 as well-preserved. New diagenetic screening analyses were carried out on specimen collections IBL, OAW, RCK (Table S1). SEM analysis showed that specimens OAW23 and RCK28 are slightly silicified, but this process is limited to the non-sampled external surface of the shell. The remainder of the Visean specimens from the UK has a well-preserved columnar layer, recording growth increments of each column. Local alteration of the fibrous secondary layer was only observed in the specimens of *Latibrachythyris* cf. *crassa*, which are mostly non-luminescent.

The specimens from the Carboniferous of Iran (labelled IR, AJ) show different degrees of preservation. With the exception of IR1073-1, IR993-307A and AJ58-37, all specimens have a well-preserved tertiary layer. The Mississippian specimens from the Mobarak Fm. (IR) have a generally well-preserved secondary layer, with only local alteration of the laminar and the fibrous secondary layers with minor laminae disruption or fibres amalgamation. They are all affected by silicification. These specimens are generally non-luminescent, except for some portions underneath the silicification which are slightly luminescent. The Moscovian specimens from the Jandaq Fm. (AJ) seem to be partially altered, but not affected by silicification. The secondary layer of the specimen AJ58-73 is slightly silicified. The specimen AJ39b-12 is altered. Specimens AJ58-73, AJ58-93 and AJ77-8 show signs of alteration in the secondary layer, while the columnar layer is well-preserved, showing growth increments in the columns. Specimens AJ58-75 and AJ58-85 have well-preserved secondary layer, which in both specimens intercalates with a well-preserved columnar layer. The specimen AJ58-93 is slightly luminescent, while the specimen AJ58-85 has some portion of the shell with a slight blue luminescence. The other samples are non-luminescent.

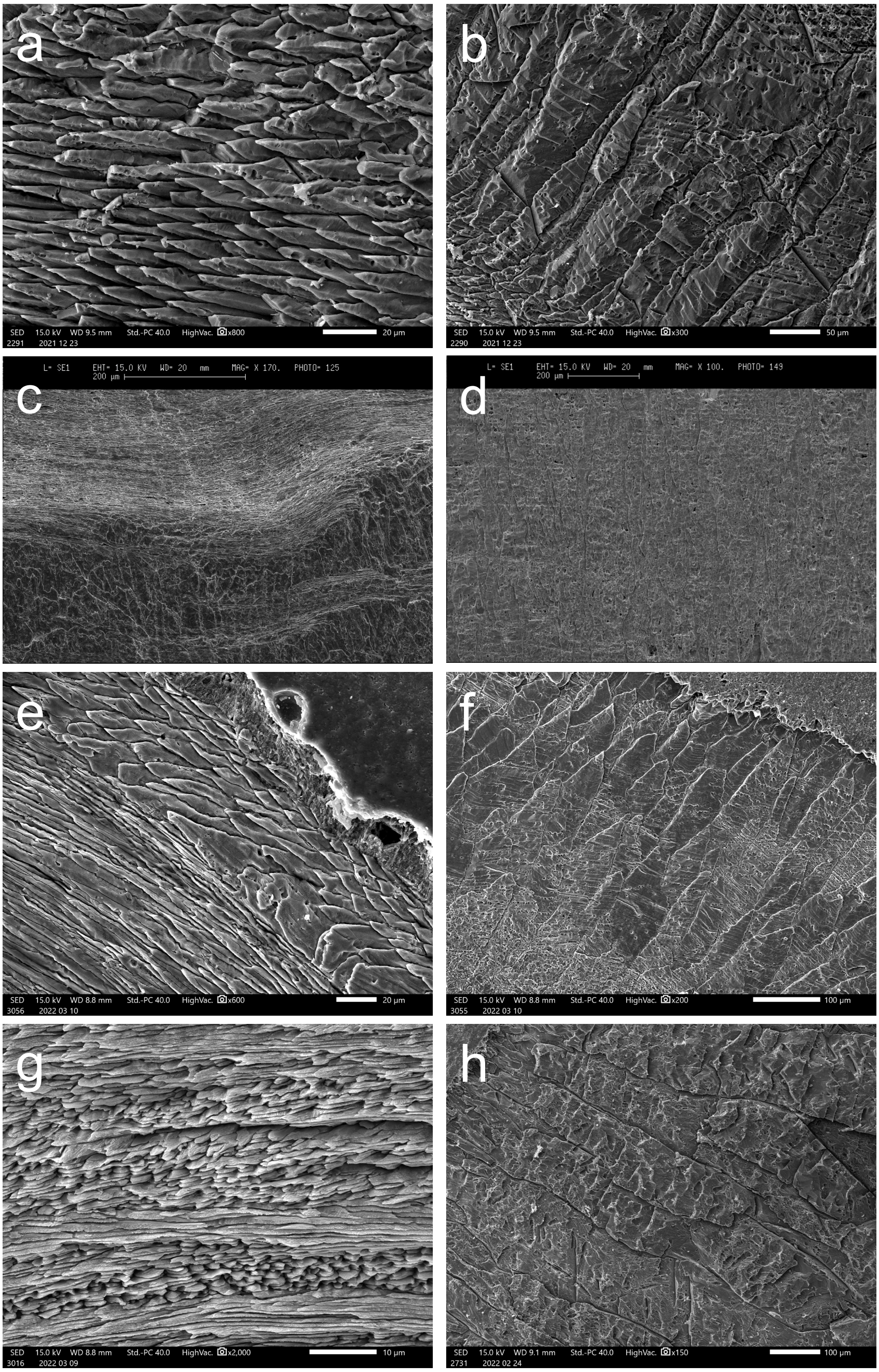

Figure S1. *Scanning electron microscope (SEM) images of selected brachiopod shells used in this study, showing good preservation of fibrous layer (a, e), laminar layer (c, g) and columnar layer (b, d, f, h) in specimens from a-b) Ible (Latibrachythyris* cf. *crassa, IBL122); c-d) Once a week (Gigantoproductus elongatus, OAW23); e-f) Ricklow (Angiospirifer sp., RCK21b); g-h) and Wadi Khawr al Jaramah (Callytharrella websteri, WKJb-2).*

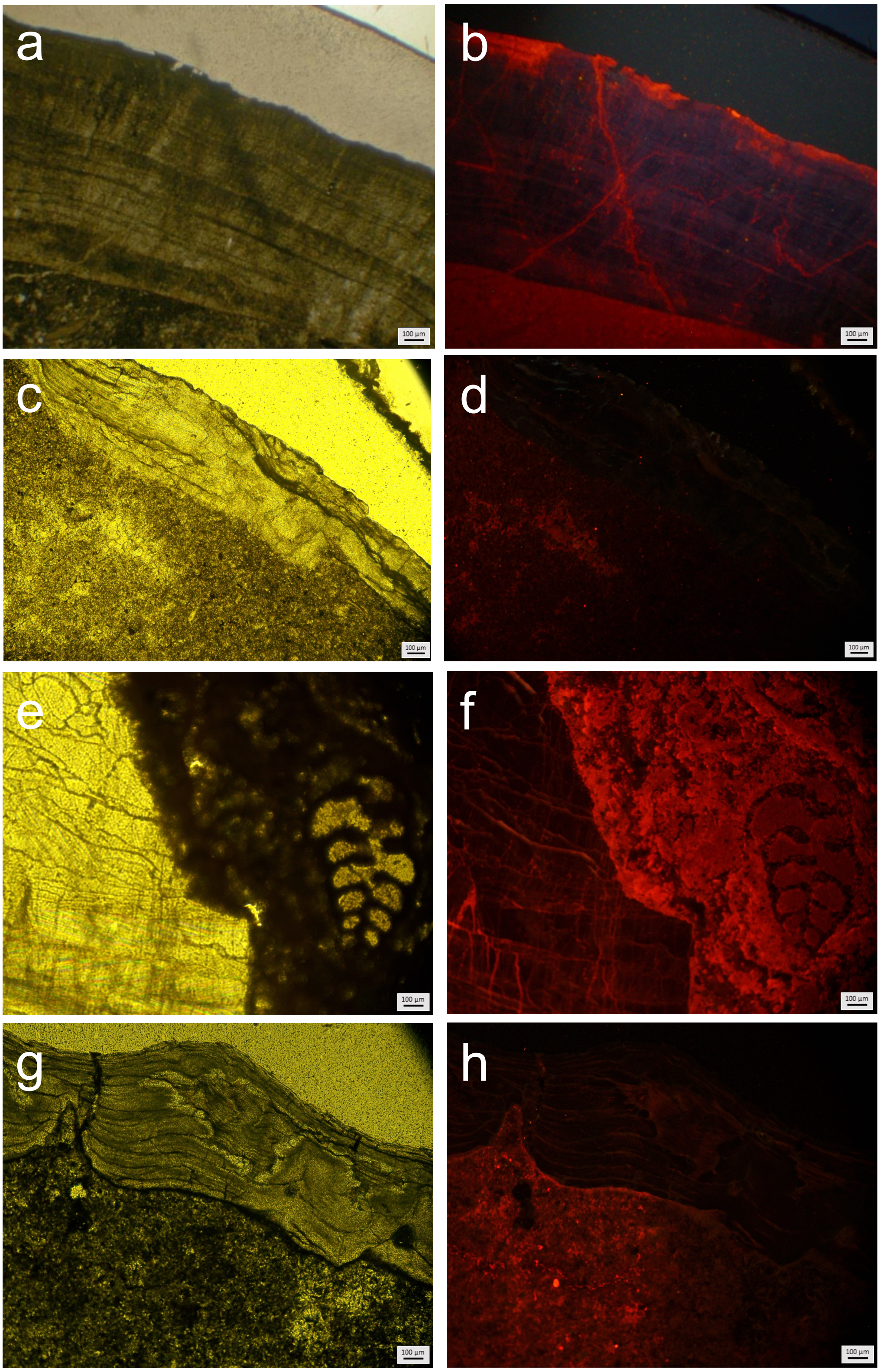

Figure S2. *Photomicrographs under parallel polarizers (left) and cathodoluminescence (right) of selected brachiopod shells used in this study. a-b) Gigantoproductus inflatus, RCK28 (Ricklow); c-d) Tomiproductus elegantulus, IR993-307A (Alborz Mts.); e-f) Choristites* aff. *mosquensis, AJ58-85 (Jandaq); g-h) Callytharrella websteri, WKJb-2 (Wadi Khawr al Jaramah).*

Brachiopod shells from the Qarari Unit, Oman (WKJ specimens) are partially silicified, but the non-silicified parts are generally very well preserved; silicification in this case led to enhanced shell preservation acting as protective sheath. The preservation of the laminae of the secondary layer of the specimen WKJb-2 is exquisite (Fig. S1g), without any trace of disruption, amalgamation or recrystallization of the laminae, and the columnar layer is also well-preserved. Other specimens with a similar preservation are WKJd-8, WKJd-40, WKJe-29B, WKJf-11 and JX3-4. In the specimen WKJf-35 both the fibrous secondary layer and the columnar tertiary layer are well preserved, with the latter recording column growth increments. The specimen WKJb-22 has a slightly altered secondary layer and an altered columnar layer; the specimen WKJf-3 has a well-preserved secondary and columnar layer, although some local alteration is present in the latter; the specimen WKJf-36 has both the secondary and the columnar layer affected by local alteration. Specimens WKJd-6 and WKJf-7 are altered with microstructural units not identifiable. Cathodoluminescence analyses showed that these shells are generally non-luminescent, independently of the microstructure preservation. Shell parts located close to the contact with silicification and/or small fractures were slightly luminescent (Fig. S2)

Chemical preservation and pristine composition of brachiopod calcite was additionally screened by Element/Ca values of samples (Data S1). Samples with Mn/Ca <500 μmol/mol were considered well-preserved. Samples with Al/Ca <500 μmol/mol were considered not contaminated from clays and enclosing sediment during sample preparation, as commonly used for screening foraminifera in palaeooceanographic studies. Our record is based on $\delta^{11}B$ that meet both thresholds, in addition to being considered well-preserved based on microstructural analyses (SEM and CL); $\delta^{11}B$ from samples with elevated Mn/Ca and Al/Ca is only included if gives comparable values to coeval samples within thresholds (in other words the inclusion of these samples does not affect the overall $\delta^{11}B$ trend), when not this data is excluded from the Carboniferous-Permian record (see Data S1, labelled 'd11B_excluded'). Most of our data was well-within both thresholds with low Mn/Ca and Al/Ca values, and overall Element/Ca composition comparable to that of recent brachiopods demonstrating the chemical integrity of our fossil specimens (Figure S3; Brand et al., 2003; Jurikova et al., 2020a). We consider these thresholds as conservative, with most samples with elevated Mn/Ca and Al/Ca not translating to biased $\delta^{11}B$ values.

The conservative selection of our thresholds is further supported by five samples with elevated Al/Ca and seven non-UK samples with elevated Mn/Ca, in both cases >500 μmol/mol but <1000 μmol/mol yielding identical $\delta^{11}B$ as their full replicates (i.e. repeat measurement of newly sampled calcite from the same shell) with low (within the threshold) Al/Ca and Mn/Ca. This indicates that variable Al/Ca and Mn/Ca (within 1000 μmol/mol) in these samples do affect their $\delta^{11}B$ values. We have thus opted for not excluding these samples from our record. Several UK specimens show elevated Mn/Ca up to ~1500 μmol/mol. These samples from the Visean outcrops of the East Neuk of Fife (Scotland), and apart from Mn/Ca do not show clear signs of alteration, and were thus also included in our record. We note that the inclusion or exclusion of these samples has no significant impact on our record and interpretations.

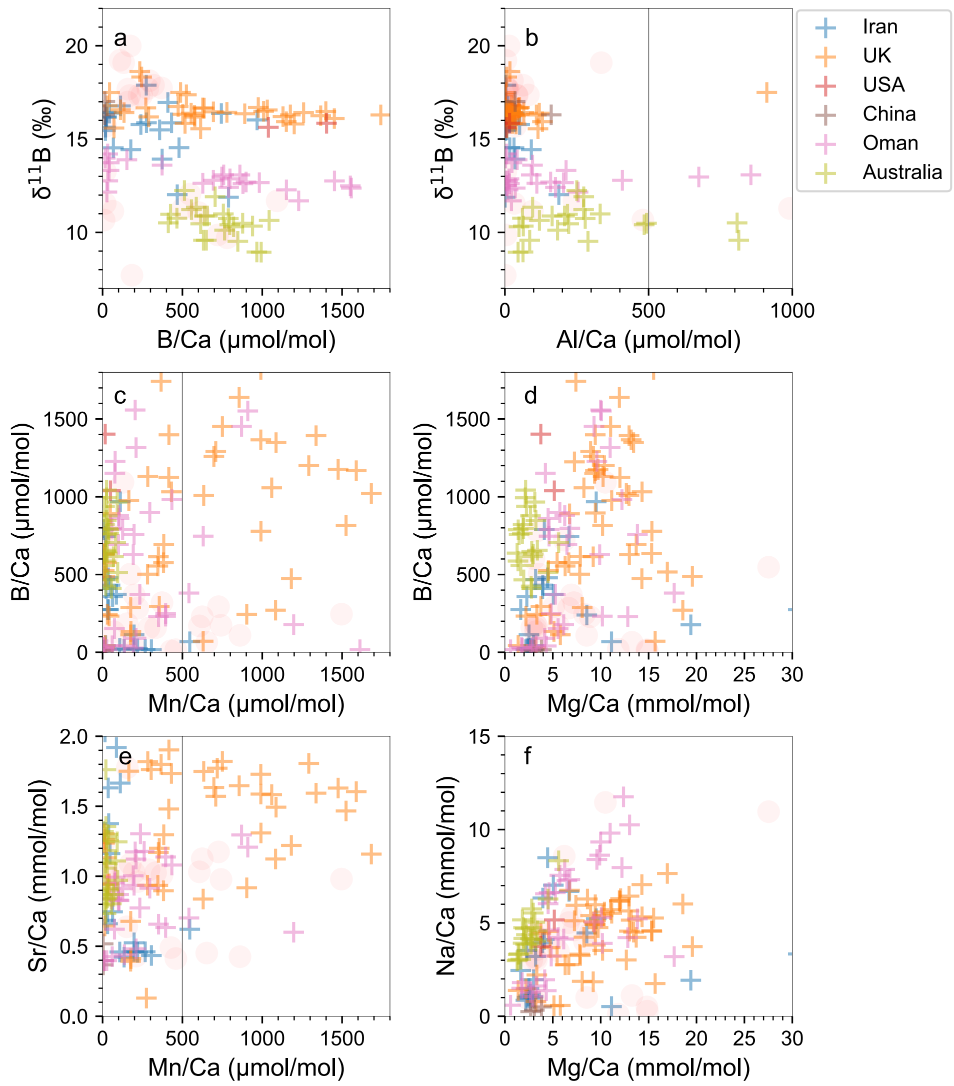

Figure S3. *Element/Ca crossplots confirming good preservation of the samples, with composition of Carboniferous-Permian fossil brachiopod shells comparable to that of extant species. Circles in panels (a) and (b) indicate excluded $\delta^{11}B$ datapoints.*

4. Vital effects and a new δ^{11}B calibration for brachiopods

Previous studies have demonstrated that brachiopods incorporate borate ion into their calcite shells, the $\delta^{11}B$ of which is closely dependant on seawater pH (Lécuyer et al., 2002, Penman et al., 2013, Jurikova et al., 2019). The $\delta^{11}B$ of brachiopods (as for the majority of calcifying organisms; see e.g. foraminifers Henehan et al., 2016) is however offset from that of ambient borate ion ($\delta^{11}B_4$), implying influences from biological regulation during biomineralisation. To correct for these 'vital effects' a calibration is needed. Explicitly, this means that while brachiopod $\delta^{11}B$ data alone tracks changes in ocean pH, a calibration is necessary to constrain the absolute ocean pH values as well as the magnitude of ocean pH change. Thus far, our knowledge of brachiopod vital effects on $\delta^{11}B$ has been limited to three different calibrations; a) a generic biogenic carbonate calibration that includes brachiopods but is not specifically aimed at brachiopods (Lécuyer et al., 2002); b) a calibration based on four different modern brachiopod species from natural settings (Penman et al. 2013); and c) a culture-based

terebratellid calibration (Jurikova et al., 2019). While these calibrations have proven invaluable for demonstrating the close pH-dependency of $\delta^{11}B$ of brachiopods, they are limited to a few species and environments only. For a more robust conversion of measured $\delta^{11}B_{brachiopod}$ values to that of $\delta^{11}B_4$ we created a new, more representative multi-species calibration (Fig. S4; Table S2). Our calibration combines previously published data and new measurements in calcitic brachiopods from different environments, thereby improving the fit, and effectively converting measured $\delta^{11}B_{brachiopod}$ to ambient $\delta^{11}B_4$ (Eq. 1).

$$\delta^{11}B_{B(OH)_4^-} = \frac{\delta^{11}B_{\text{brachiopod}} - 5.0936}{0.7735} \qquad \text{(Eq. 1)}$$

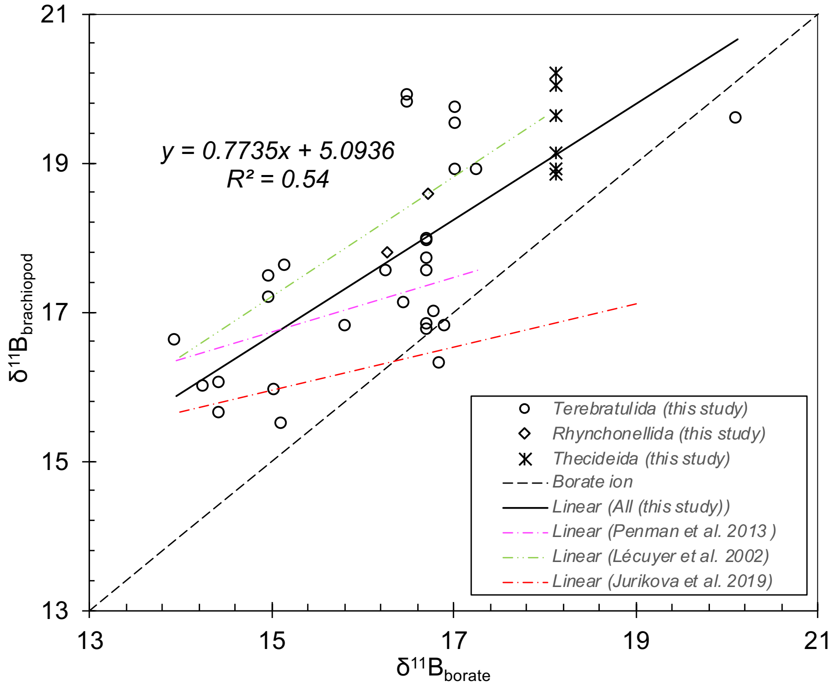

Figure S4. *New brachiopod vital effect calibration (solid black line) for converting* $\delta^{11}B_{brachiopod}$ *to* $\delta^{11}B_4$*. Shown are also existing brachiopod calibrations from Lécuyer et al. (2002), Penman et al. (2013), and Jurikova et al. (2019).*

Even though our calibration provides a substantial improvement over previously available calibrations, we note that scatter remains in the data that deserves attention (Fig. 3). We attribute this scatter to seasonal fluctuations in seawater pH at the sample collection site, which is not unexpected for natural sampling sites (e.g. as opposed to culturing studies). Despite our estimation of aqueous $\delta^{11}B_4$ being based, to our best knowledge, on the most representative available mean seawater pH at a given site (Table S2), variations in local seawater pH are challenging to account for in the $\delta^{11}B_4$ calculation and cannot be excluded. This might, logically, lead to the question as to whether such fluctuation in seawater pH may not be biasing brachiopod-based $\delta^{11}B$ records from Earth's past. Any such potential bias can be avoided by basing pH reconstruction not on single $\delta^{11}B$ data points, but instead an interpolated best-fit pH reconstruction through multiple $\delta^{11}B$ data points from a given site (see Section 6 below), as well as additionally assuring that any $\delta^{11}B$ trends interpreted are reproducible between sites – as shown in our study. The comprehensive nature of our $\delta^{11}B$ dataset combined with the conservative statistical modelling employed to derive seawater pH thus provides confidence that our reconstructed trends are representative and not affected by potential uncertainties arising from vital effect calibration, variations between individuals and/or at sites.

No.	Species	Order	Location	Depth	Collected	$\delta^{11}B_{brachiopod}$ (‰)	± 2SD (‰)	$\delta^{11}B_{borate}$ (‰)
1	*Notosaria nigricans*	Rhynchonellida	Doubtful Sound-Tricky Cove	20 m	recent	18.58	0.20	16.72
2	*Notosaria nigricans*	Rhynchonellida	Pacific Ocean, New Zealand	5 m	-	17.79	0.30	16.27
3	*Calloria inconspicua*	Terebratulida	Doubtful Sound-Tricky Cove	20 m	recent	16.75	0.20	16.72
4	*Calloria inconspicua*	Terebratulida	Doubtful Sound-Tricky Cove	20 m	recent	16.82	0.20	16.72
5	*Terebratella sanguinea*	Terebratulida	Doubtful Sound-Tricky Cove	20 m	recent	17.55	0.20	16.72
6	*Terebratella sanguinea*	Terebratulida	Doubtful Sound-Tricky Cove	20 m	recent	17.94	0.20	16.72
7	*Terebratella sanguinea*	Terebratulida	Doubtful Sound-Tricky Cove	20 m	recent	17.96	0.20	16.72
8	*Terebratella sanguinea*	Terebratulida	Doubtful Sound-Tricky Cove	20 m	recent	17.71	0.20	16.72
9	*Dallinella occidentalis*	Terebratulida	Catalina Island, CA	91 m		17.60	0.33	15.15
10	*Dallinella occidentalis*	Terebratulida	Catalina Island, CA	37 m		17.00	0.33	16.80
11	*Fallax neocaledonensis*	Terebratulida	Pacific Ocean, New Caledonia	430 m	-	16.81	0.30	16.93
12	*Frenulina sanguinolenta*	Terebratulida	Punta Engano, Philippines	25 m	2018	19.58	0.20	20.13
13	*Gryphus vitreus*	Terebratulida	Mediterranean Sea, Marseille	150 m	-	18.90	0.30	17.04
14	*Gryphus vitreus*	Terebratulida	Mediterranean Sea, Marseille	150 m	-	19.72	0.30	17.04
15	*Gryphus vitreus*	Terebratulida	Mediterranean Sea, Marseille	150 m	-	19.52	0.30	17.04
16	*Laqueus californicus*	Terebratulida	Catalina Island, CA	64 m		16.80	0.38	15.82
17	*Laqueus californicus*	Terebratulida	Refugio Canyon, CA	238 m		16.00	0.33	14.25
18	*Magellania venosa*	Terebratulida	Argentina	108 m		16.30	0.35	16.85
19	*Magellania venosa*	Terebratulida	Comau Fjord, Chile	20 m	recent	15.95	0.20	15.05
20	*Magellania venosa*	Terebratulida	Argentina	71 m		17.10	0.33	16.46
21	*Terebratalia coreanica*	Terebratulida	Yellow Sea, China	40 m	2010	15.50	0.20	15.12
22	*Terebratalia transversa*	Terebratulida	Pacific Ocean, Vancouver	intertidal	-	17.17	0.30	15.00
23	*Terebratalia transversa*	Terebratulida	Pacific Ocean, Vancouver	intertidal	-	17.47	0.30	15.00
24	*Terebratalia transversa*	Terebratulida	San Pedro, CA	5 m		18.90	0.38	17.27
25	*Terebratalia transversa*	Terebratulida	The Narrows, Tacoma, WA	5 m		16.60	0.27	13.95
26	*Terebratalia transversa*	Terebratulida	San Juan Islands-Channel	64 m	recent	15.64	0.20	14.44
27	*Terebratalia transversa*	Terebratulida	San Juan Islands-Channel	64 m	recent	16.04	0.20	14.44
28	*Terebratulina hataiana*	Terebratulida	East China Sea, China	130 m	2012	19.89	0.20	16.51
29	*Terebratulina hataiana*	Terebratulida	East China Sea, China	130 m	2012	19.81	0.20	16.51
30	*Waltonia inorspicua*	Terebratulida	Pacific Ocean, New Zealand	intertidal	-	17.55	0.30	16.27
31	*Pajaudina atlantica*	Thecideida	La Palma, Canary Islands	20 m	recent	18.86	0.20	18.13
32	*Pajaudina atlantica*	Thecideida	La Palma, Canary Islands	20 m	recent	18.93	0.20	18.13
33	*Pajaudina atlantica*	Thecideida	La Palma, Canary Islands	20 m	recent	19.13	0.20	18.13
34	*Pajaudina atlantica*	Thecideida	La Palma, Canary Islands	20 m	recent	19.63	0.20	18.13
35	*Pajaudina atlantica*	Thecideida	La Palma, Canary Islands	20 m	recent	20.04	0.20	18.13
36	*Pajaudina atlantica*	Thecideida	La Palma, Canary Islands	20 m	recent	20.21	0.20	18.13
37	*Lingula sp.*	Lingulida	South China Sea		recent	13.23	0.20	14.74
38	*Lingula sp.*	Lingulida	South China Sea		recent	12.97	0.20	14.74

Reference[a] - boron isotope values

Reference[b] - seawater physico-chemical parameters

[c]IPACOA - IOOS Partners Across Coasts Ocean Acidification Data Portal

Table S2. *Boron isotope composition of modern brachiopods and corresponding seawater physico-chemical parameters used to derive the new calibration for brachiopod vital effects (continued on the next page).*

No.	Species	Reference[a]	pH	T	Sal	Reference[b]
1	*Notosaria nigricans*	This study	8.04	15.0	34.0	Jackson Bay, NZOA-ON data from NIWA
2	*Notosaria nigricans*	Lécuyer et al. (2002)	8.00	14.0	34.0	Chatham Islands, NZOA-ON data from NIWA
3	*Calloria inconspicua*	This study	8.04	15.0	34.0	Jackson Bay, NZOA-ON data from NIWA
4	*Calloria inconspicua*	This study	8.04	15.0	34.0	Jackson Bay, NZOA-ON data from NIWA
5	*Terebratella sanguinea*	This study	8.04	15.0	34.0	Jackson Bay, NZOA-ON data from NIWA
6	*Terebratella sanguinea*	This study	8.04	15.0	34.0	Jackson Bay, NZOA-ON data from NIWA
7	*Terebratella sanguinea*	This study	8.04	15.0	34.0	Jackson Bay, NZOA-ON data from NIWA
8	*Terebratella sanguinea*	This study	8.04	15.0	34.0	Jackson Bay, NZOA-ON data from NIWA
9	*Dallinella occidentalis*	Penman et al. (2013)	7.89	10.3	33.4	CCHDO P02 station 117
10	*Dallinella occidentalis*	Penman et al. (2013)	8.05	15.0	33.5	CCHDO P02 station 117
11	*Fallax neocaledonensis*	Lécuyer et al. (2002)	7.93	24.0	35.0	Wu et al. (2018)
12	*Frenulina sanguinolenta*	This study	8.18	29.1	34.5	Hayasaka et al. (1982) Avg. all stations at 20 m
13	*Gryphus vitreus*	Lécuyer et al. (2002)	8.06	14.0	38.0	Hemming et al. (2017) and others
14	*Gryphus vitreus*	Lécuyer et al. (2002)	8.06	14.0	38.0	Hemming et al. (2017) and others
15	*Gryphus vitreus*	Lécuyer et al. (2002)	8.06	14.0	38.0	Hemming et al. (2017) and others
16	*Laqueus californicus*	Penman et al. (2013)	7.97	11.7	33.6	CCHDO P02 station 117
17	*Laqueus californicus*	Penman et al. (2013)	7.74	8.1	34.1	CCHDO P02 station 117
18	*Magellania venosa*	Penman et al. (2013)	8.15	7.2	33.9	CCHDOA17 station 3
19	*Magellania venosa*	Jurikova et al. (2019)	7.90	10.0	30.0	Jurikova et al. (2019)
20	*Magellania venosa*	Penman et al. (2013)	8.18	1.8	33.9	GEOSECS
21	*Terebratalia coreanica*	This study	7.90	10.0	32.0	Zhai (2018)
22	*Terebratalia transversa*	Lécuyer et al. (2002)	7.95	6.0	29.0	IPACOA[c]
23	*Terebratalia transversa*	Lécuyer et al. (2002)	7.95	6.0	29.0	IPACOA[c]
24	*Terebratalia transversa*	Penman et al. (2013)	8.06	18.6	33.3	Berelson (1985)
25	*Terebratalia transversa*	Penman et al. (2013)	7.71	7.7	29.6	Simone Alin (NOAA) pers. comm.
26	*Terebratalia transversa*	This study	7.80	9.0	30.0	Murray et al. (2015)
27	*Terebratalia transversa*	This study	7.80	9.0	30.0	Murray et al. (2015)
28	*Terebratulina hataiana*	This study	7.95	20.0	34.0	Chou et al. (2013), Chung-Chi Chen et al. (2020)
29	*Terebratulina hataiana*	This study	7.95	20.0	34.0	Chou et al. (2013), Chung-Chi Chen et al. (2020)
30	*Waltonia inorspicua*	Lécuyer et al. (2002)	8.00	14.0	34.0	Chatham Islands, NZOA-ON data from NIWA
31	*Pajaudina atlantica*	Jurikova et al. (2019)	8.10	21.0	36.0	Jurikova et al. (2019)
32	*Pajaudina atlantica*	This study	8.10	21.0	36.0	Jurikova et al. (2019)
33	*Pajaudina atlantica*	This study	8.10	21.0	36.0	Jurikova et al. (2019)
34	*Pajaudina atlantica*	This study	8.10	21.0	36.0	Jurikova et al. (2019)
35	*Pajaudina atlantica*	This study	8.10	21.0	36.0	Jurikova et al. (2019)
36	*Pajaudina atlantica*	This study	8.10	21.0	36.0	Jurikova et al. (2019)
37	*Lingula sp.*	This study	7.70	20.0	34.5	Jurikova et al. (2022)
38	*Lingula sp.*	This study	7.70	20.0	34.5	Jurikova et al. (2022)

Reference[a] - boron isotope values

Reference[b] - seawater physico-chemical parameters

[c]IPACOA - IOOS Partners Across Coasts Ocean Acidification Data Portal

Table S2. *Continued.*

5. $^{87}Sr/^{86}Sr$-time curve modelling

Background and assumptions

The $^{87}Sr/^{86}Sr$ ratio of marine (biogenic) carbonates can be used to estimate the age of marine sediments since the $^{87}Sr/^{86}Sr$ value of Sr dissolved in the oceans has varied predictably through time. Therefore, the construction of our $^{87}Sr/^{86}Sr$-time curve lies on the assumption that the Sr isotopic composition of ocean water is homogeneous in a given time interval because the residence time of this element in the seawater (~10^6 years) is longer than the ocean mixing time (10^3 years) (Brand et al., 2003; Korte and Ullmann, 2018).

To build our curve we use the principle of correlation, rather than dating by the traditional approach entailing the comparison with a reference curve (McArthur et al., 2020). Here the variation of the marine $^{87}Sr/^{86}Sr$ ratio during a specific geological time interval is included in a statistical model where $^{87}Sr/^{86}Sr$ values and time are correlated by an unknown function; despite the real factors that control the secular variations in marine Sr budget being related to geological and climatic evolution of the Earth system. We use a local regression (LOESS, which is a generalization of the LOWESS) to build the curve (Fig. S5). When data are fitted using the LOESS, points located closer to a given estimation have greater weights than those located farther. Since $^{87}Sr/^{86}Sr$ data of similar age have a greater likelihood of giving the same result, the local regression approach describes well changes in $^{87}Sr/^{86}Sr$ over time.

Unfortunately, samples often occur in localities where dating is limited and ages poorly constrained. Numerical ages are often assigned on the basis of assumptions about sedimentary rates and duration of biozones where radiometric dating is not available. Here we use a randomness number generation within the LOESS regression mathematical model to solve the numerical ages of samples with known $^{87}Sr/^{86}Sr$ (but unknown numerical age), instead of assigning an arbitrary numerical age.

The two variables included in the model – i.e. the $^{87}Sr/^{86}Sr$ ratio and the age of samples – are constrained with a different degree of knowledge. Owing to the increasing precision of $^{87}Sr/^{86}Sr$ measurements and our improved ability to discriminate between pristine and diagenetic signatures, the model uncertainties are largely related to the error associated with dating of the samples and/or to the sampling density along a stratigraphic section (Garbelli et al., 2019; Wang et al., 2021; Wierzbowski et al., 2017). This is particularly true for the sections where no radiometric data are available and the ages are solely assigned on the basis of biozones and stratigraphic depth (Wang et al., 2018).

To assemble a coherent $^{87}Sr/^{86}Sr$-age model, the implemented method accounts for the following points: (i) it is not possible to know the precise age of the samples and the uncertainty is difficult to estimate; (ii) the ages of samples can only be constrained by biozones, magnetostratigraphy, or radiometric age, when they are available in the section under study; it must be also accounted that biozones can be diachronous, adding, thus, uncertainty to ages; (iii) the possible theoretical distribution of the ages along a section can be estimated after a careful evaluation of sample distribution and sedimentological characteristics of a section; (iv) the implemented method aims to build a model coherent with the stratigraphic position of the samples, thus the information related to the position must be used to constrain the generated age model.

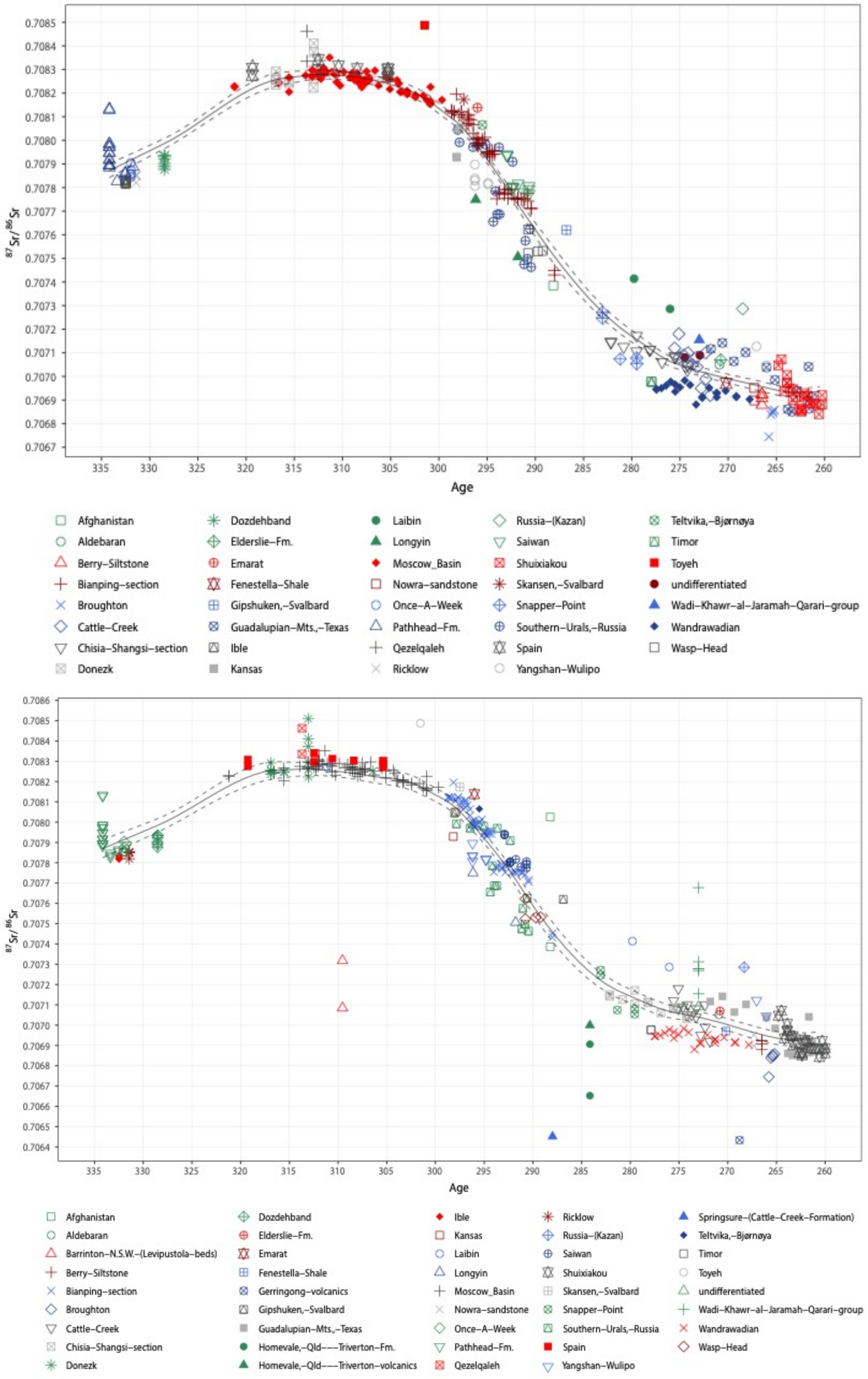

Figure S5. *Our compiled Carboniferous-Permian $^{87}Sr/^{86}Sr$ curve built using LOESS; colour-coded according to formations from which brachiopod samples came from. The top panel shows the here-used $^{87}Sr/^{86}Sr$ curve after rejection of anomalous values; the bottom panel shows all data compiled including anomalous values (see also discussion in section 'Datasets employed' below). Line shows the mean fit with 95% confidence intervals.*

At the end of the process, this approach allows the determination of sample age and its confidence interval. The criteria to select the best model are established and, moreover, the $^{87}Sr/^{86}Sr$-age model generated must be coherent with other proposed models, when available.

Method structure

To build the $^{87}Sr/^{86}Sr$-age model, the starting point is to define an age interval and the possible statistical distribution of ages within (Fig. S6); we refer to these two parameters as boundary conditions. These boundary conditions must be precisely selected, accounting for samples spacing and sedimentological evidences. If there is no clear bias in the sampling distribution along the section, or clear indication of changes in the sedimentary rates, we suggest using a uniform distribution (Fig. S6a). Alternatively, if this does not apply a skewed distribution may be considered (Fig. S6b).

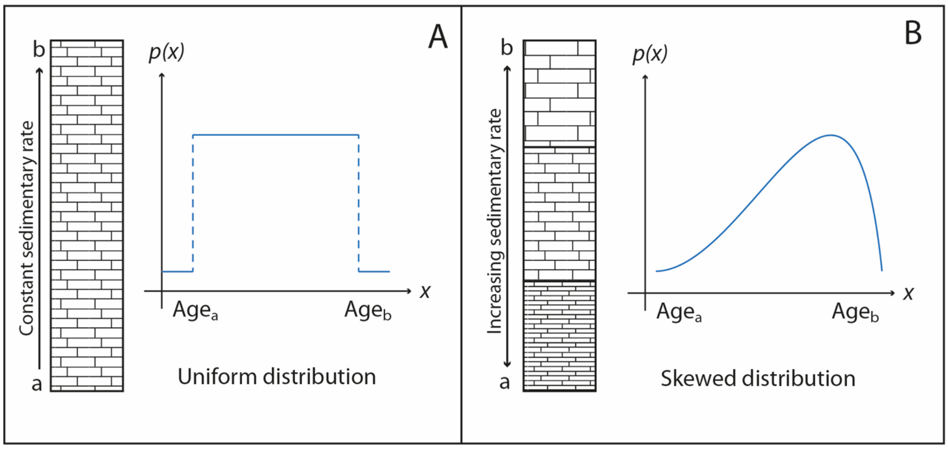

Figure S6. *Idealized statistical distribution of samples age for stratigraphic sections with different sedimentation rates; the samples collection is hypothesized to be homogeneous; (A) the sedimentary rate is constant and all the ages have equal probability to be represented by the samples (Uniform distribution); (B) the sedimentary rate increases from the bottom to the top; the youngest age has more probability to be represented by the samples (Skewed distribution).*

After carefully evaluating the boundary conditions, the ages are randomly generated for each $^{87}Sr/^{86}Sr$ value and constrained to follow the stratigraphic position of samples, so that the LOESS model can be computed (Fig. S7). The simulation consists of generating different sets of ages, which follow the initial boundary conditions, and thus gaining different LOESS models, which are referred to as simulations. The selection of a different statistical distribution can lead to different results of the simulations (Fig. S8). Selecting the SPAN to be used in the model generation is also another important step of the analysis, since it can significantly affect the final results (Fig. S9). Here, the smoothing parameter has been selected using the bias-corrected Akaike information criterion (AICc) (Hurvich et al., 1998) and, thus, every simulation has a different SPAN. The frequency distribution of the confidence intervals of the simulations is used to select the simulations to be employed in the estimation of ages means and their confidence interval (CI). At this point, the underlying ages of simulations with confidence interval values within the 95% CI are extracted, and each age mean and its confidence interval is calculated. In addition, it is possible to extract the mean fitted trend in its confidence interval (Fig. S10). An unpolished R code to perform the analyses in R is available on request from the authors.

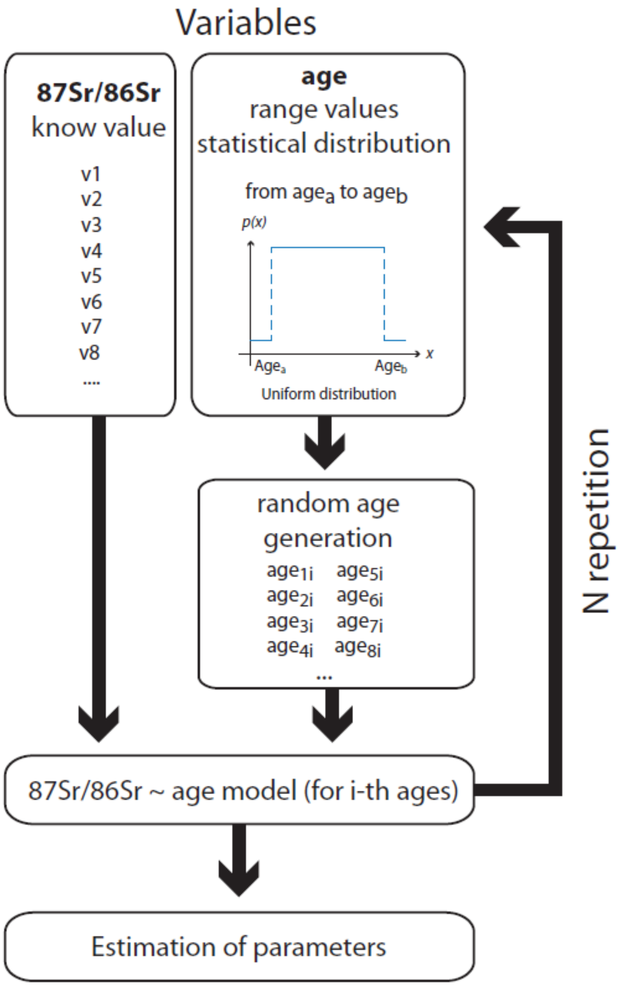

Figure S7. *Schematic diagram of the approach exemplified step by step.*

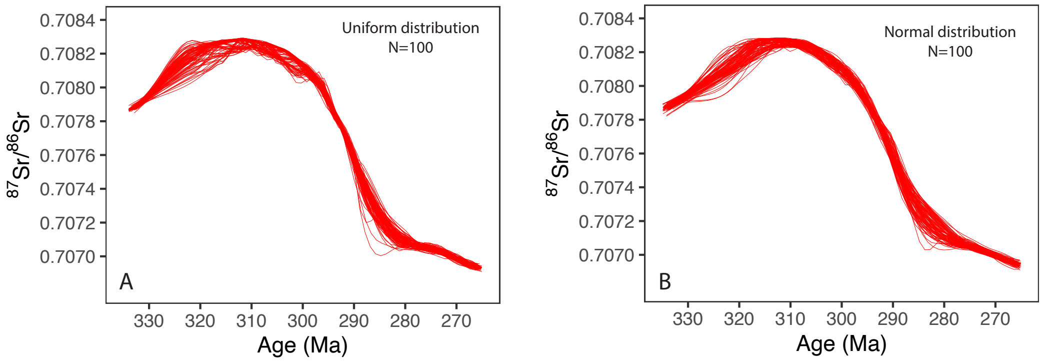

Figure S8. *$^{87}Sr/^{86}Sr$-time models employing uniform and normal distribution of ages for late Carboniferous to early Permian: (A) uniform distribution; (B) normal distribution; the exemplified simulations are generated including the data from Garbelli et al. (2019), Korte et al. (2006), Morante (1996), Brand et al. (2012) and Garbelli et al. (2016).*

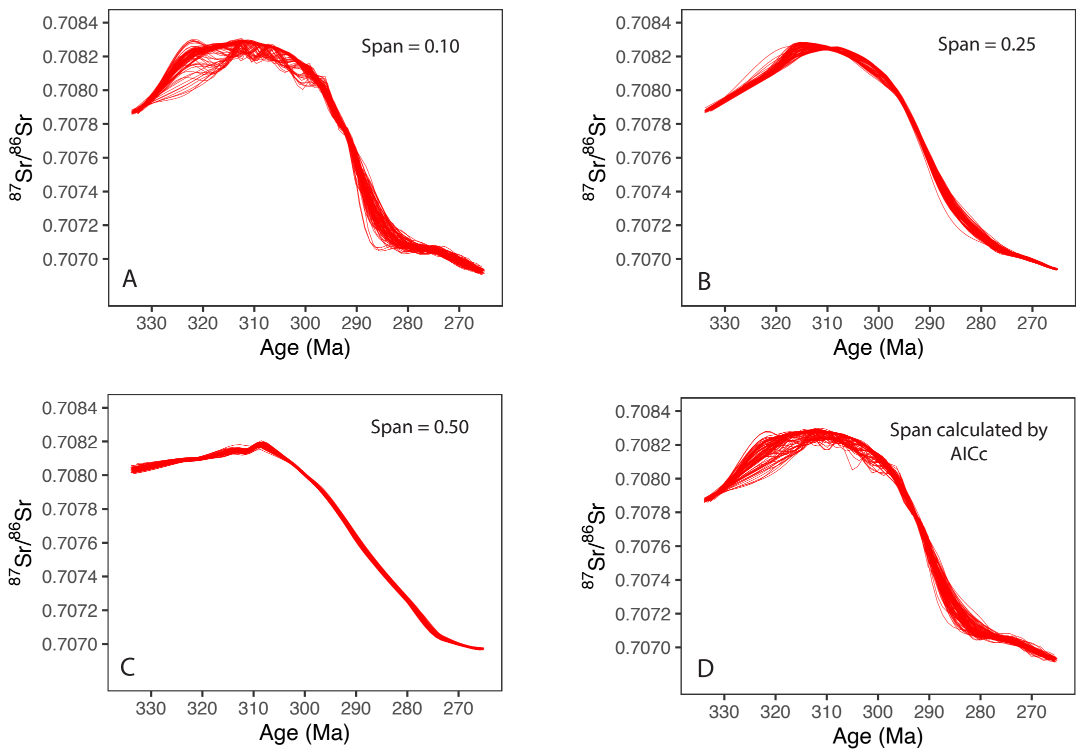

Figure S9. *Underlying simulations for the late Carboniferous and early Permian. Panels A–C show three different simulations with a fixed SPAN value selected by the user, each leading to a different result and a simulation with the SPAN selected following the AICc (D); in panel D each line is calculated selecting optimized SPAN values for each of the models.*

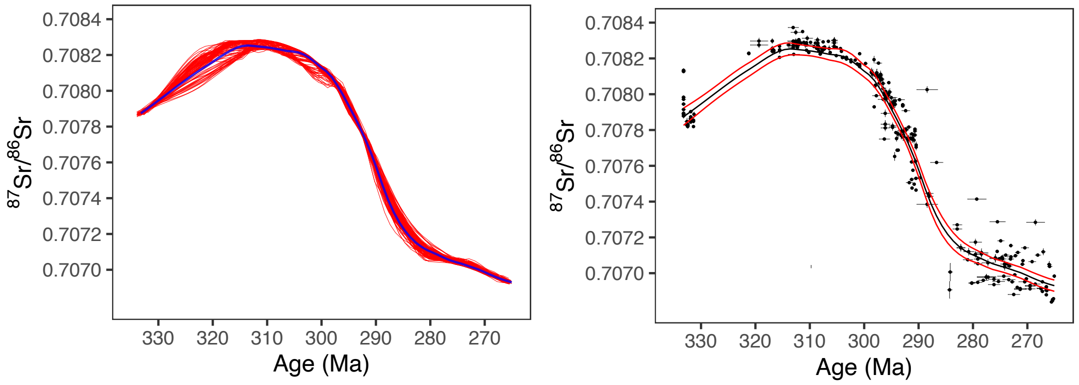

Figure S10. *(A) mean fit (blue line) calculated on the best simulated models (red lines); (B) mean fit, its confidence interval and points used to build the model; samples ages shows the confidence intervals (95%) calculated from this final model. The modelling is based on the late Carboniferous and early Permian data included in Figure S8.*

Datasets employed

We built the $^{87}Sr/^{86}Sr$ curve using only values measured on calcite brachiopods shells. Our database includes new data measurements as well as previous data published from the literature, which are summarized in Table S3. We carefully reviewed the data previously published to assess if the Sr isotope values are enough preserved to be included in our model, or rejected because they are not representative of compositions derived from precipitation in the marine environment. We took in consideration the data available from published literature, such as

information about shell microstructure, trace element composition and stable isotopes of oxygen and carbon, when available. Moreover, if $^{87}Sr/^{86}Sr$ values of some samples scattered widely away from coeval material from the same locality, we excluded the samples in absence of clear evidence of preservation (Fig. S5). For example, the sample E-5-735 from Morante (1999) gives the two least radiogenic values recorded in the entire Permian (0.70646; 0.70651). Despite the fact that the Ingelara formation was deposited around a time where relatively low values of $^{87}Sr/^{86}Sr$ were recorded (Garbelli et al., 2019), these results stand out as anomalous and not convincing for at least two reasons. Firstly, their $\delta^{13}C$ values are lower if compared with other brachiopods from these localities. Secondly, Morante (1999) used cathodoluminescence as the main screening tool for alteration. Unfortunately, this tool alone has proven to be insufficient since brachiopods can be naturally enriched in Fe, which dampens the luminescence and could lead to inappropriate evaluation. Indeed, coeval brachiopods from the Sydney Basin are naturally enriched in Fe, and other elements than Mn and Fe seems to be more diagnostic to assess the preservation (Garbelli et al., 2019). Therefore, we excluded these two values from our model. Similarly, some of the data of Veizer et al. (1999), show values much more radiogenic than samples from coeval ages (e.g. compare his samples 240B and 242A with 246B). These samples have also $\delta^{18}O$ values more negative and were thus excluded (Fig. S5).

After excluding $^{87}Sr/^{86}Sr$ values considered not preserved, the age interval of samples is assigned based on the biozone. Here ages were assigned using conodont, fusulinids and palynological biozones. Radiometric ages are rarely available at the sampling sites. Sometimes radiometric ages are available for the formation where samples have been collected, and they provide an additional constraint for the interval dating. We used the latest version of the Permian timescale to constrain ages of biozones (https://permian.stratigraphy.org/timescale; Permian Timescale, 2022). Palynological Zones were employed to assign ages to the samples of Morante (1999), following the latest radiometric calibration (Laurie et al., 2016), adjusted to the latest version of the Permian Timescale (2022). Our database also includes many samples from the Carboniferous of the Russian Platform. Here the age assignment is based on Russian Platform subdivisions of Cohen et al. (2013), updated to the Geologic Time Scale 2022 (Angiolini et al., 2021).

**Author(s)**	**Year**	**Period/Epoch/Stage covered**	**Region**
Veizer & Compston	1974		
Popp et al.	1986	Pennsylvanian/Cisuralian	
Denison et al.	1993	Wuchiapingian	Iran
Veizer et al.	1999	Carboniferous	
Morante	1999	Permian	Australia
Korte et al.	2006	Permian	
Angiolini et al.	2008	Sakmarian	Oman
Brand et al.	2010		
Brand et al.	2012	Latest Changhsingian	Italy
Garbelli et al.	2016	Changhsingian	Tibet
Garbelli et al.	2019	Cisuralian/Guadalupian	Australia
Wang et al.	2021	Permian	China

Table S3. *Datasets employed to build the $^{87}Sr/^{86}Sr$ – time model.*

To evaluate the robustness of this approach we compare published ages of a marine sediment core assigned based on stratigraphic position (from Hodell et al., 1991) to ages generated for

the same core from $^{87}Sr/^{86}Sr$ using our randomisation experiment (Fig. S11). For this, we deliberately choose a well-constrained marine sediment core with robust age model (instead of a comparatively less well characterised Palaeozoic outcrop), which provides a more adequate (and challenging) test for our approach. Our results (Fig. S11) demonstrate that this approach generates highly reliable ages, even when minimal chronostratigraphic information is available.

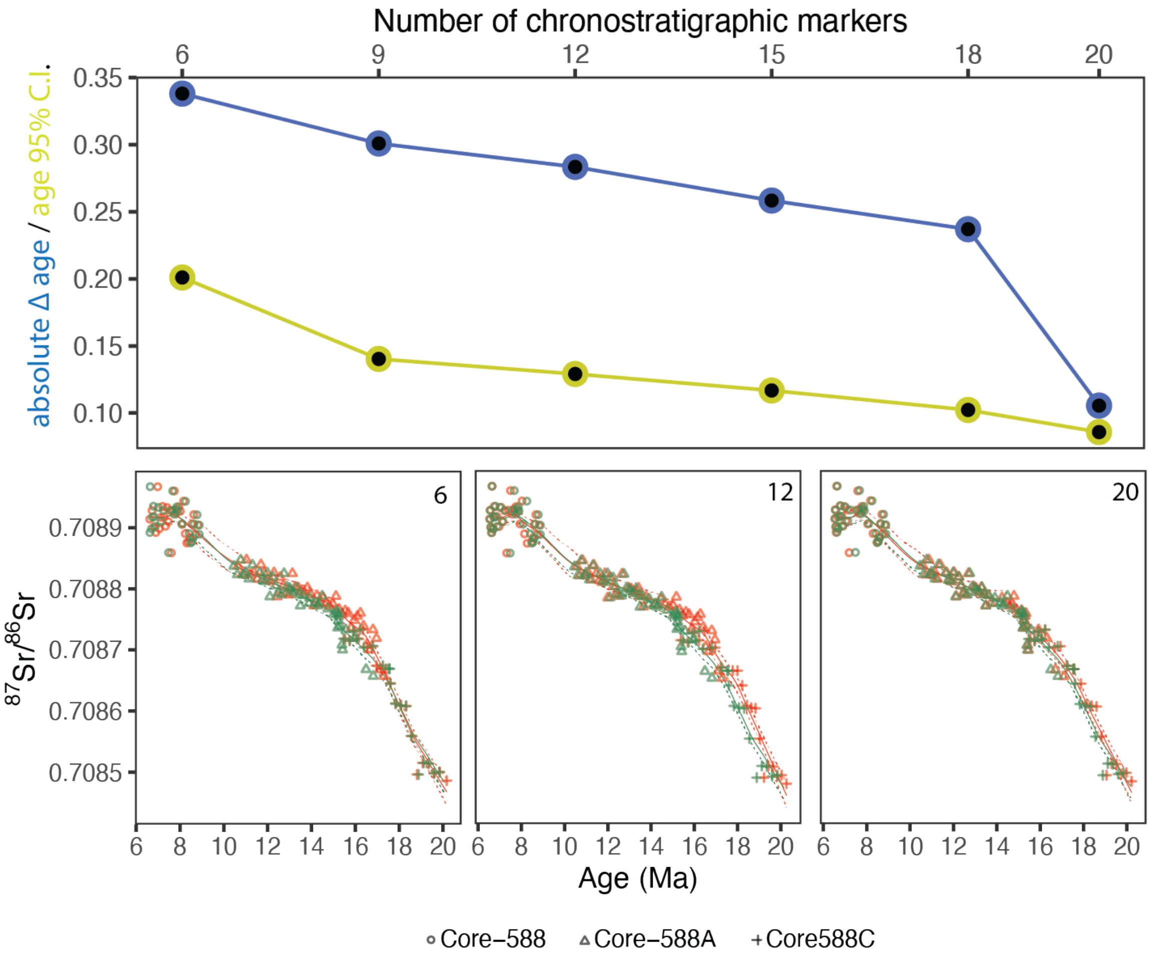

Figure S11. *(Top) The relative difference between published ages of a marine sediment core (data from Hodell et al., 1991) and ages derived for the same core using our $^{87}Sr/^{86}Sr$ randomisation approach plotted as a function of number of chronostratigraphic markers used. The ages calculated in Hodell et al. (1991) are based on 34 age-depth control points; our approach produces within error identical ages when using at least 20 age-depth controls and reasonable age estimates with as little as 6 age-depth points (i.e. those bounding the holes 588, 588A and 588C). (Bottom) $^{87}Sr/^{86}Sr$-age plots showing in green the marine Sr-trend plotted against stratigraphic ages and in orange against here-calculated ages using our approach. Panels show increasing number of chronostratigraphic markers used (6, 12, 20). Note that already at 6 chronostratigraphic constraints our model produced a shape highly consistent with that of Hodell et al. (1991), demonstrating that our approach provides reliable ages even when minimal constraints are used. Line shows the mean fit with 95% confidence intervals.*

6. Modelling of $\delta^{11}B_{sw}$, ocean pH and atmospheric CO_2

Calculation of pH from boron isotopes requires a number of ancillary parameters: a species calibration (to convert $\delta^{11}B_{brachiopod}$ to $\delta^{11}B_{borate}$ or $\delta^{11}B_4$), the boron isotope composition of seawater ($\delta^{11}B_{sw}$), the fractionation factor (ε), and the equilibrium constant (pK^*_B), which itself requires temperature, pressure, salinity, and ocean ionic composition, in particular calcium $[Ca]_{sw}$ and magnesium $[Mg]_{sw}$ concentrations. Most of these parameters can be reasonably estimated from data collected in this study (species calibration, temperature and pressure), data from pre-existing work (ε, $[Ca]_{sw}$ and $[Mg]_{sw}$), or assumed with an appropriate uncertainty (salinity). We use ε from Klochko et al. (2006) and Nir et al. (2015). We take Carboniferous $[Ca]_{sw}$ and $[Mg]_{sw}$ from Holt et al. (2014) and Permian seawater from Horita et al. (2002) and Lowenstein et al. (2003). Temperature is derived from $\delta^{18}O$ data, calculated as: $15.7 - 4.36 \times (\delta^{18}O_{measured} - \delta^{18}O_{sw}) + 0.12 \times (\delta^{18}O_{measured} - \delta^{18}O_{sw})^2$, applying a constant value of –1‰ for $\delta^{18}O_{sw}$ (see below section *Constraints and their justification* for discussion of the justification of this approach).

$\delta^{11}B_{sw}$, however, is currently unconstrained for this time period, and has a strong relationship with both pH and ΔpH calculated from boron isotopes. A very broad constraint can be placed on $\delta^{11}B_{sw}$ by exploiting the sigmoidal relationship between $\delta^{11}B_4$ and pH, which is a result of the fractionation factor ε. ε is understood to be constant (or near constant) and there are two key estimates of its value: 27.2±0.6 ‰ (Klochko et al., 2006) and 26.0±1.0 ‰ (Nir et al., 2015). From the measured $\delta^{11}B_{brachiopod}$ and species calibration, we are able to calculate the values that $\delta^{11}B_{sw}$ could take on without violating the underlying mechanics of the boron-pH proxy. This gives a plausible $\delta^{11}B_{sw}$ range for each measured $\delta^{11}B$ value which is wide but robust.

Our approach to improve constraints on $\delta^{11}B_{sw}$ is to combine the broad constraint described above with the $^{87}Sr/^{86}Sr$ record, which is used to guide our understanding of change in seawater boron composition. $^{87}Sr/^{86}Sr$ and $\delta^{11}B_{sw}$ share major controls (weathering and hydrothermal inputs, Figure S12; Greenop et al., 2017; Whiteford et al., 2023), so we expect them to evolve along similar pathways. To translate our generated $^{87}Sr/^{86}Sr$ record to $\delta^{11}B_{sw}$ space, we first interpolate the $^{87}Sr/^{86}Sr$, with smoothing used to ensure that the timescale of change is not inconsistent with the long residence time of boron (~10 Myr). The interpolated record of $^{87}Sr/^{86}Sr$ is normalised, such that the range is from 0 to –1. This relative change is then scaled into permille by multiplying by a randomly drawn value within a prescribed range, and adding a randomly drawn value from the possible initial $\delta^{11}B_{sw}$ (Fig. S12).

The method described above allows us to generate possible evolutions of $\delta^{11}B_{sw}$ and calculate plausible evolutions of ocean pH. Calculation of atmospheric CO_2 from ocean pH requires knowledge of a second carbonate system parameter. Our approach to constrain the second carbonate system parameter (DIC), as mentioned in the main text, is based on a null hypothesis that assumes invariant CO_2. We calculate the change in DIC that would be required to maintain invariant atmospheric CO_2 concentration, the apply a randomly drawn multiplier to our hypothetical DIC evolution, which allows the impact of changing pH on CO_2 to be damped, amplified, or completely inverted by changing DIC. These hypothetical pH and DIC evolutions are validated by ensuring both atmospheric CO_2 and Ω stay within reasonable limits (see below).

We use a Markov Chain Monte Carlo (MCMC) approach to integrate these various constraints. On each iteration, uncertainty in all values is simulated by making small changes to the measured $\delta^{11}B$, $\delta^{11}B_{sw}$ scaling factor, initial $\delta^{11}B_{sw}$, and fraction of CO_2 change explained by

change in DIC. pH and CO_2 are then calculated, and samples are accepted or rejected using the Metropolis-Hastings algorithm. The probability of acceptance depends on the $\delta^{11}B_{sw}$, pH, CO_2, and saturation state. $\delta^{11}B_{sw}$, saturation state, and CO_2 and provide a binary constraint: if the value is not within the plausible range (10–10,000 ppm for CO_2, 1-15 for saturation state, and determined by the sigmoidal relationship of $\delta^{11}B_{sw}$ and pH for $\delta^{11}B_{sw}$), then the sample is rejected. The basis for these chosen ranges are discussed in the section below. We run the algorithm to generate 11,111 samples, discarding the first 10% for burn in, and calculate posterior metrics on the remaining 10,000 samples.

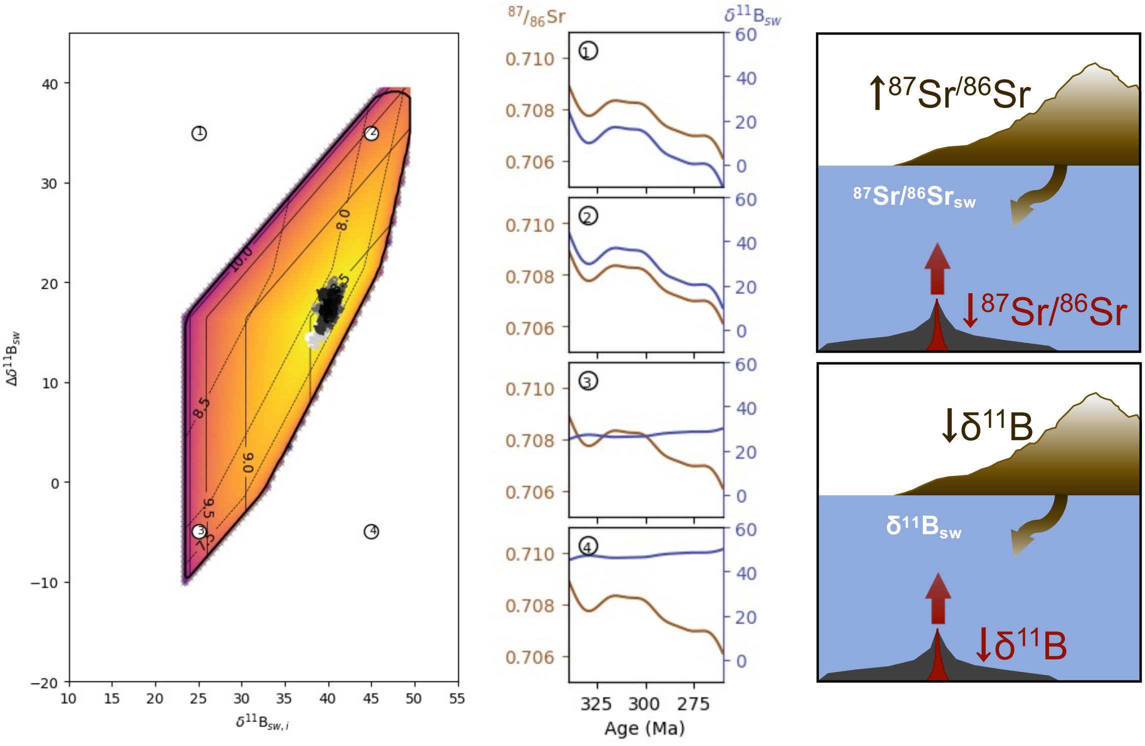

Figure S12. *$^{87}Sr/^{86}Sr$ evolution over time informs the nature of $\delta^{11}B_{sw}$ evolution. The simulated $\delta^{11}B_{sw}$ curves are drawn by normalizing the strontium curve to have a range of 1, and an initial value of 0 (as described in this section), then scaling and shifting this curve to translate the normalized values into $\delta^{11}B_{sw}$. The two parameters (scaling and shifting) form the axes of the leftmost subplot above. Examples of how these parameters translate $^{87}Sr/^{86}Sr$ to $\delta^{11}B_{sw}$ are shown in subplots 1 through 4. For instance, subplot 1 shows a low initial $\delta^{11}B_{sw}$ with a strong positive relationship to $^{87}Sr/^{86}Sr$, while subplot 4 shows a high initial $\delta^{11}B_{sw}$ with a weak negative relationship to $^{87}Sr/^{86}Sr$. The range which is valid for our dataset is shown by the outlined region in the lefthand panel, within which contours for minimum pH (dotted contours) and maximum pH (solid contours) are shown. Yellow colours indicate regions of high probability (where both minimum and maximum pH are close to 8.0), and purple colours indicate regions of low probability. Crosses show the trajectory of our MCMC algorithm which optimises toward pH = 8.0, with white crosses indicating the earliest accepted samples and black crosses the latest accepted samples.*

Constraints and their justification

In addition to the data presented here or available from prior work, there are several constraints here that we need to calculate or impose. Here we discuss considerations involved in deriving these constraints. In general, where assumptions have been made, we take care to choose those which are conservative in terms of our estimates of atmospheric CO_2 change to ensure any trends we reconstruct are robust.

As discussed, calculation of pH and CO_2 concentration requires an estimate of temperature. Here we use $\delta^{18}O$ data, assuming a constant $\delta^{18}O_{sw.}$ Constant $\delta^{18}O_{sw}$ is a necessary simplification as Carboniferous-Permian $\delta^{18}O_{sw}$ trends are unknown. The choice of $\delta^{18}O_{sw}$ might potentially bias calculated temperatures – in this case giving (if anything) a cooler bias in Early Permian temperatures (i.e. an underestimate of end-LPIA warming). Underestimation of temperature minimises atmospheric CO_2 concentration due to the sensitivity of CO_2 solubility to temperature. In this sense, our reconstructed CO_2 change is conservative. To illustrate the effect of post-LPIA warming on our calculation of atmospheric CO_2 we, in addition, provide a second reconstruction which considers an SST increase of ~9 °C from Late Carboniferous to Early Permian, and could be thus considered more representative.

Quantifying the carbonate system as described produces possible evolutions of pH, atmospheric CO_2 concentration, and calcite saturation state. These are parameters for which we have some sense of the reasonable space in which they must sit (in other words, a prior). For pH we prescribe a prior of 8±1 unit at 1 standard deviation, which covers two orders of magnitude in hydrogen ion [H^+] concentration, and makes acceptance of samples with unrealistic pH values exceptionally unlikely. Our broad understanding of pH at this time is that, if anything it is likely to be at 8 or lower.

For atmospheric CO_2, we set the plausible range to 10–10,000 ppm. Note the plausible CO_2 range used here is deliberately broad and purely theoretical; we do not imply that at any time CO_2 might have been as low as 10 ppm or as high as 10,000 ppm, but that CO_2 must have been somewhere within this broad range.

Finally, for the saturation state of calcite, we set the plausible range to anywhere between 1 and 15. A saturation state of below 1 results in no preservation of carbonate (which can not have occurred due to the presence of our samples), and a saturation state of greater than 15 for any significant time would cause very widespread inorganic carbonate precipitation including in much of the deep ocean, which is also thought not to have occurred. Accurate calculation of saturation state requires quantification of the seawater calcium concentration. Seawater calcium concentration has been determined from fluid inclusions (Holt et al. 2014, Horita et al. 2002, and Lowenstein et al. 2003), but we acknowledge that such estimations are inherently uncertain. Therefore, we allow a degree of flexibility in the calcium concentration. This is achieved by first calculating the saturation state using the standard Markov Chain approach, and if the saturation state is only slightly greater than 15 or less than 1, the calcium concentration is adjusted up to 50% away from the value suggested by fluid inclusions. This assumption is generous with regards to the uncertainty imparted by limitations on our understanding of seawater calcium concentration at this time.

Our approach to constrain the second carbonate system parameter (DIC) is based on a null hypothesis that assumes invariant CO_2. Here, we test an alternate scenario that instead starts with the assumption of invariant saturation (Fig. S13). The latter generates similar trajectories

and relative change in CO_2, underpinning our findings of major CO_2 release at the Asselian-Sakmarian boundary, although the reconstructed CO_2 change is magnified.

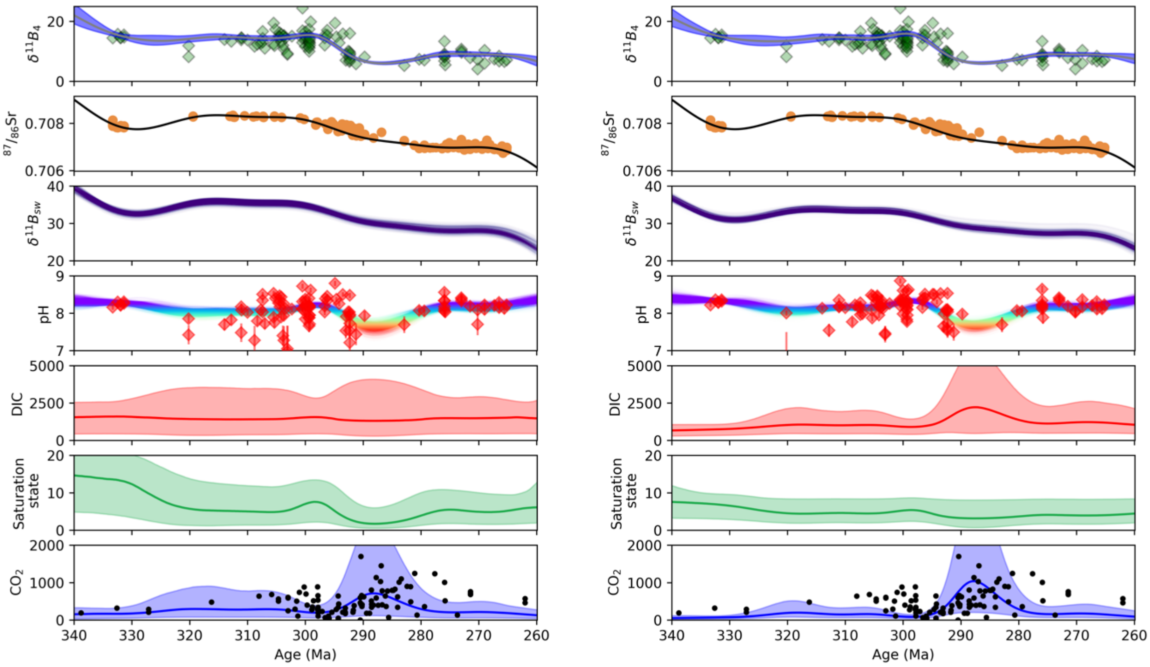

Figure S13. *(Left) Main (reference) reconstruction based on the initial assumption of invariant CO_2, and (right) an alternate scenario based on the initial assuption of invariant saturation stage. Green diamonds show $\delta^{11}B_4$ data (with 2SD), orange circles $^{87}Sr/^{86}Sr$ data, red diamonds sample-paired reconstructed pH, and black dots previously published CO_2 estimates, for comparison. The interpolated lines show median values with 95% confidence interval envelope. Individual lines in the $\delta^{11}B_{sw}$ and pH panels (colour-coded according to pH) correspond to one iteration of the algorithm.*

We constrain DIC by applying multipliers to our hypothetical DIC evolution (based on invariant CO_2), allowing the impact of changing pH on CO_2 to be damped, amplified, or completely inverted by changing DIC, with the final chosen DIC evolution validated by ensuring both atmospheric CO_2 and Ω stay within reasonable limits. Here, we illustrate the sensitivity of pH and CO_2 to different DIC by comparing selected interpolations where DIC fraction is high (>0.0; DIC change is acting to mitigate CO_2 change) and where DIC fraction is low (<0.0; DIC change is strenghtening CO_2 change; Fig. S14). Even when DIC fraction is high, there is a pronounced increase in atmospheric CO_2, demonstrating that it is very unlikely that the pH change at ~294 Ma can be explained by anything other than a sizeable increase in atmospheric CO_2.

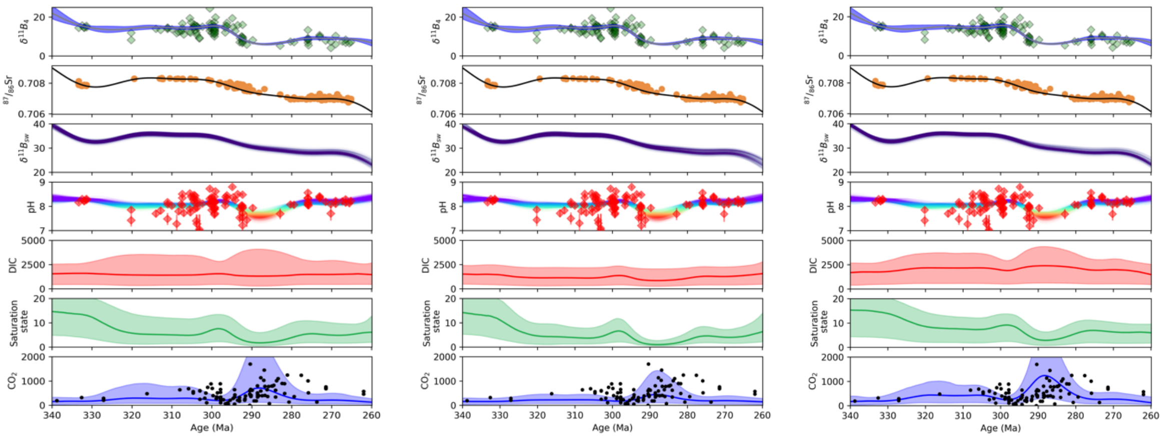

Figure S14. *(Left) Main (reference) reconstruction, and selected interpolations with (middle) high DIC fraction (>0.0; DIC change is acting to mitigate CO_2 change), and (right) low DIC fraction (<0.0; DIC change is strenghtening CO_2 change). All scenarios demonstrate a sizeable increase in atmospheric CO_2 about 294 Ma at the Asselian-Sakmarian boundary. The interpolated lines show median values with 95% confidence interval envelope. Individual lines in the $\delta^{11}B_{sw}$ and pH panels (colour-coded according to pH) correspond to one iteration of the algorithm.*

7. Palaeo-artistic reconstruction of the Late Palaeozoic world

Accurate illustration of the Late Palaeozoic world was created based on our geological and palaeontological observations, and the palaeoclimatic and palaeoenvironmental reconstructions from our study. Further supporting evidence came from consultation of museum exhibits and literature, particularly relevant were publications by Aretz et al. (2020), Augusta & Burian (1956), Benton (2014), Baumgarten et al. (2016), DiMichele et al. (2001, 2005, 2006), Dunne et al. (2018), Falcon-Lang (2003), Gornitz (2008), Lescaze & Ford (2017), Šimůnek & Martínek (2009), and Shi et al. (2021). The illustration is a digital painting with all subjects drawn by hand, posteriorly edited in Photoshop applying filters and digital Photoshop tools to acquire photorealism and colour uniformity.

References

Angiolini L. (2007). Quantitative palaeoecology in the *Pachycyrtella* Bed, Early Permian of Interior Oman. Palaeoworld 16, 233–245, https://doi.org/10.1016/j.palwor.2007.05.019.

Angiolini L., Stephenson M.H., Leven E.J. (2006). Correlation of the Lower Permian Surface Saiwan Formation and subsurface Haushi Limestone, Central Oman. GeoArabia, 11, 17–37, https://doi.org/10.2113/geoarabia110317.

Angiolini L., Balini M., Garzanti E., Nicora A., Tintori A. (2003) Gondwanan deglaciation and opening of Neotethys: the Al-Khlata and Saiwan formations of Interior Oman. Palaeogeogr. Palaeoclimatol. Palaeoecol. 196, 99–123, https://doi.org/10.1016/S0031-0182(03)00315-8.

Angiolini L., Darbyshire D.P.F., Stephenson M.H., Leng M.J., Brewer T.S., Berra F., Jadoul F. (2008) Lower Permian brachiopods from Oman: their potential as climatic proxies. Trans. Roy. Sco. Edinburgh 98, 327–344, https://doi.org/10.1017/S1755691008075634.

Angiolini L., Stephenson M.H., Leng M.J., Jadoul F., Millward D., Aldridge A., Andrews J.E., Chenery S., Williams G. (2012). Heterogeneity, cyclicity and diagenesis in a Mississippian

brachiopod shell of palaeoequatorial Britain. Terra Nova 24, 16–26, https://doi.org/10.1111/j.1365-3121.2011.01032.x.

Angiolini L., Crippa G., Azmy K., Capitani G., Confalonieri G., Della Porta G., Griesshaber E., Harper D.A.T., Leng M.J., Nolan L., Orlandi M., Posenato R., Schmahl W.W., Banks V.J., Stephenson M.H. (2019) The giants of the phylum brachiopoda: A matter of diet? Paleontology 62, 889–917, https://doi.org/10.5061/dryad.m42t6tm.

Aretz M., Herbig H. G., Wang X. D., Gradstein F. M., Agterberg F. P., Ogg J. G. (2020). Chapter 23 – The Carboniferous Period. In Geologic Time Scale 2020, Elsevier (pp. 811–874), https://doi.org/10.1016/B978-0-12-824360-2.00023-1.

Augusta J. and Burian Z. (1956). Prehistoric animals. London Spring Books.

Benton M. J. (2014). Vertebrate palaeontology. 4th Edition, John Wiley & Sons.

Baumgardner Jr R. W., DiMichele W. A., de Siqueira Vieira N. (2016). An early Permian coastal flora dominated by *Germaropteris martinsii* from basinal sediments in the Midland Basin, West Texas. Palaeogeogr. Palaeoclimatol. Palaeoecol. 459, 409–422. https://doi.org/10.1016/j.palaeo.2016.07.024.

Bahrammanesh M., Angiolini L., Antonelli A.A., Aghababalou B., Gaetani M. (2011) Tournaisian (Mississippian) brachiopods from the Mobarak Formation, North Iran. GeoArabia 16, 129–192, https://doi.org/10.2113/geoarabia1603129.

Berra F., Zanchi A., Angiolini L., Vachard D., Vezzoli G., Zanchetta S., Bergomi M., Hamid Reza Javadi, Meyssam Kouhpeima (2017) The Upper Palaeozoic Godar–E-Siah Complex of Jandaq: Evidence and significance of a North Palaeotethyan succession In Central Iran. J. Asian Earth Sci. 138, 272–290, https://doi.org/10.2113/geoarabia1603129.

Berelson W. (1985) Studies of Water Column Mixing and Benthic Exchange of Nutrients. Carbon and Radon in the Southern California Borderland. University of Southern California, 219 pp.

Brand U., Logan A., Hiller N., Richardson J. (2003) Geochemistry of modern brachiopods: applications and implications for oceanography and paleoceanography. Chem. Geol. 198, 305–334, https://doi.org/10.1016/s0009-2541(03)00032-9.

Brand U., Azmy K., Tazawa J.-I., Sano H., Buhl D. Hydrothermal diagenesis of Paleozoic seamount carbonate components. Chem. Geol. 278, 173–185 (2010). https://doi.org/10.1016/j.chemgeo.2010.09.010

Brand U, Posenato R., Came R., Affek H., Angiolini L., Azmy K. Farabegoli E. The end-Permian mass extinction: A rapid volcanic CO_2 and CH_4-climatic catastrophe. Chem. Geol. 322-323, 121–144 (2012). https://doi.org/10.1016/j.chemgeo.2012.06.015

Brenckle P.A., Gaetani M., Angiolini L., Bahrammanesh M. (2009) Refinements in biostratigraphy, chronostratigraphy, and paleogeography of the Mississippian (Lower Carboniferous) Mobarak Formation, Alborz Mountains, Iran. GeoArabia 14, 42–78, https://doi.org/10.2113/geoarabia140343.

Carniti A.P., Della Porta G., Banks V., Stephenson M.H., Angiolini L. (2023) Revisiting the upper Visean mud mounds from Derbyshire (UK): the role of brachiopods in their growth. Facies 69, 9, https://doi.org/10.1007/s10347-023-00663-w.

Chou W.-C., Gong G.-C., Hung C.-C., Wu Y.-H. (2013) Carbonate mineral saturation states in the East China Sea: present conditions and future scenarios. Biogeosciences 10, 6453–6467, https://doi.org/10.5194/bg-10-6453-2013.

Chung-Chi Chen, Gong G.-C., Chou W.C., Shiah F.-K. (2020) Hypoxia in autumn of the East China Sea. Mar. Pollut. Bull. 152, 110875, https://doi.org/10.1016/j.marpolbul.2019.110875.

DiMichele, W. A., Pfefferkorn, H. W., & Gastaldo, R. A. (2001). Response of Late Carboniferous and Early Permian plant communities to climate change. Annual Review of

Earth and Planetary Sciences, 29(1), 461–487, https://doi.org/10.1146/annurev.earth.29.1.461.
DiMichele W. A., Gastaldo R. A., Pfefferkorn H. W. (2005). Plant biodiversity partitioning in the Late Carboniferous and Early Permian and its implications for ecosystem assembly. Proceedings of the California Academy of Sciences 56, 24–41.
DiMichele W. A., Tabor N. J., Chaney D. S., Nelson W. J. (2006). From wetlands to wet spots: environmental tracking and the fate of Carboniferous elements in Early Permian tropical floras. Geological Society of America, Special Paper 399, 223–248, https://doi.org/10.1130/2006.2399(11).
Dunne E. M., Close R. A., Button D. J., Brocklehurst N., Cashmore D. D., Lloyd G. T., Butler R. J. (2018). Diversity change during the rise of tetrapods and the impact of the 'Carboniferous rainforest collapse'. Proceedings of the Royal Society B: Biological Sciences, 285, 20172730, https://doi.org/10.1098/rspb.2017.2730.
Falcon-Lang H. J. (2003). Late Carboniferous tropical dryland vegetation in an alluvial-plain setting, Joggins, Nova Scotia, Canada. Palaios, 18, 197–211, https://doi.org/10.1669/0883-1351(2003)018<0197:LCTDVI>2.0.CO;2.
Fielding C.R., Al-Rubaii, Walton E.K. (1988) Deltaic sedimentation in an unstable tectonic environment - the Lower Limestone Group (Lower Carboniferous) of East Fife, Scotland. Geol. Mag. 125, 241-255, https://doi.org/10.1017/S0016756800010189.
Garbelli C., Angiolini L., Brand U., Shen S-Z., Jadoul F., Posenato R., Azmy K., Cao C-Q. (2016) Neotethys seawater chemistry and temperature at the dawn of the end Permian mass extinction. Gondwana Res. 35, 272–285, https://doi.org/10.1016/j.gr.2015.05.012.
Garbelli C., Shen S.Z., Immenhauser A., Brand U., Buhl D., Wang W.Q., Zhang H., Shi G.R. (2019) Timing of Early and Middle Permian deglaciation of the southern hemisphere: Brachiopod-based $^{87}Sr/^{86}Sr$ calibration. Earth Planet. Sci. Lett. 516, 122–135, https://doi.org/10.1016/j.epsl.2019.03.039.
Garbelli C., Cipriani A., Brand U., Lugli F., Posenato R. (2022a) Strontium isotope stratigraphic insights on the end-Permian mass extinction and the Permian-Triassic boundary in the Dolomites (Italy). Chem. Geol. 605, 120946, https://doi.org/10.1016/j.chemgeo.2022.120946.
Garbelli C., Angiolini L., Posenato R., Harper E.M., Lamare M.D., Shi G.R., Shen S.-Z. (2022b). Isotopic time-series ($\delta^{13}C$ and $\delta^{18}O$) obtained from the columnar layer of Permian brachiopod shells are a reliable archive of seasonal temperature variations. Palaeogeogr. Palaeoclimatol. Palaeoecol. 607, 111264, https://doi.org/10.1016/j.palaeo.2022.111264.
Greenop R., Hain M. P., Sosdian S.M., Oliver K.I.C., Goodwin P., Chalk T.B., Lear C.H., Wilson P.A., Foster G.L. (2017) A record of Neogene seawater $\delta^{11}B$ reconstructed from paired $\delta^{11}B$ analyses on benthic and planktic foraminifera. Clim. Past 13, 149–170, https://doi.org/10.5194/cp-13-149-2017.
Gornitz V. (2008). Encyclopedia of paleoclimatology and ancient environments (Ed.). Springer Science & Business Media, https://doi.org/10.1007/978-1-4020-4411-3.
Grossman E.L., Zhang C., Yancey T.E. (1991) Stable-isotope stratigraphy of brachiopods from Pennsylvanian shales in Texas. Geol. Soc. Amer. Bull. 103, 953–965, https://doi.org/10.1016/j.palaeo.2008.03.053.
Grossman E.L., Mii H.-S., Yancey T.E. (1993) Stable isotopes in late Pennsylvanian brachiopods from the United States: implications for Carboniferous paleoceanography. Geol. Soc. Amer. Bull. 105, 1284–1296, https://doi.org/10.1130/0016-7606(1993)105<1284:SIILPB>2.3.CO;2.
Grossman E.L., Yancey T.E., Jones T.E., Bruckschen P., Chuvashov B., Mazzullo S.J., Mii H.-S. (2008) Glaciation, aridification, and carbon sequestration in the Permo-Carboniferous:

The isotopic record from low latitudes. Palaeogeogr. Palaeoclimatol. Palaeoecol. 268, 222–233, https://doi.org/10.1016/j.palaeo.2008.03.053.

Gutjahr M., Bordier L., Douville E., Farmer J., Foster G. L., Hathorne E. C., Hönisch B., Lemarchand D., Louvat P., McCulloch M., Noireaux J., Pallavicini N., Rae J.W.B., Rodushkin I., Roux P., Stewart J.A., Thil F., You C.-F. (2020). Sub-Permil Interlaboratory Consistency for Solution-Based Boron Isotope Analyses on Marine Carbonates. Geostandards and Geoanalytical Research 45, 59–75, https://doi.org/10.1111/ggr.12364.

Hayasaka S., Saisho T., Kakinuma Y., Shinomiya A., Oki K., Hamada T., Tanabe K., Kanie Y., Hattori M., Vande Vusse F., Alcala L., Cordero Jr. P.A., Cabrera J.J., Garcia R.G. (1982) Field Study on the Habitat of Nautilus in the Environs of Cebu and Negros Islands, the Philippines. Mem. Kagoshima Univ. Res. Center S. Pac. 3, 67–116.

Hemming M.P., Kaiser J., Heywood K.J., Bakker D.C.E., Boutin J., Shitashima K., Lee G., Legge O., Onken R. (2017) Measuring pH variability using an experimental sensor on an underwater glider. Ocean Sci. 13, 427–442, https://doi.org/10.5194/os-13-427-2017.

Henehan M.J., Foster G.L., Bostock H.C., Greenop R., Marshall B.J., Wilson P.A. (2016) A new boron isotope-pH calibration for *Orbulina universa*, with implications for understanding and accounting for 'vital effects. Earth Planet Sci. Lett. 454, 282–292, https://doi.org/10.1016/j.epsl.2016.09.024.

Hodell D.A., Mueller P.A., Garrido J.R. (1991) Variations in the strontium isotopic composition of seawater during the Neogene. Geology 19, 24–27, https://doi.org/10.1130/0091-7613(1991)019<0024:VITSIC>2.3.CO;2.

Holt N.M., García-Veigas J., Lowenstein T.K., Giles P.S., Williams-Stroud S. (2014) The major-ion composition of Carboniferous seawater. Geochim. Cosmochim. Acta 134, 317-334, https://doi.org/10.1016/j.gca.2014.03.009.

Horita J., Zimmermann H., Holland H.D. (2002) Chemical evolution of seawater during the Phanerozoic: Implications from the record of marine evaporites. Geochim. Cosmochim. Acta 66, 3733-3756, https://doi.org/10.1016/S0016-7037(01)00884-5.

Hurvich C.M., Simonoff J.S., Tsai C.L. (2002) Smoothing parameter selection in nonparametric regression using an improved Akaike information criterion. J. R. Stat. Soc., B: Stat. 60, 271–293, https://doi.org/10.1111/1467-9868.00125.

Joachimski M.M., Simon L., van Geldern R., Lécuyer C. (2005) Boron isotope geochemistry of Paleozoic brachiopod calcite: Implications for a secular change in the boron isotope geochemistry of seawater over the Phanerozoic. Geochim. Cosmochim. Acta 69, 4035–4044, https://doi.org/10.1016/j.gca.2004.11.017.

Jurikova H., Liebetrau V., Gutjahr M., Rollion-Bard C., Hu M. Y., Krause S., Henkel D., Hiebenthal C., Schmidt M., Laudien J. and Eisenhauer A. (2019) Boron isotope systematics of cultured brachiopod calcite: response to acidification, vital effects and implications for palaeo-pH reconstruction. Geochim. Cosmochim. Acta 248, 370–386, https://doi.org/10.1016/j.gca.2019.01.015.

Jurikova H., Ippach M., Liebetrau V., Gutjahr M. Krause S., Büsse S., Gorb S.N., Henkel D., Hiebenthal C., Schmidt M., Leipe T., Laudien J., Eisenhauer A. (2020a) Incorporation of minor and trace elements into cultured brachiopods: Implications for proxy application with new insights from a biomineralisation model. Geochim. Cosmochim. Acta 286, 418–440, https://doi.org/10.1016/j.gca.2020.07.026.

Jurikova H., Gutjahr M., Wallmann K., Flögel S., Liebetrau V., Posenato R., Angiolini L., Garbelli C., Brand U., Wiedenbeck M., and Eisenhauer A. (2020b) Permian-Triassic mass extinction pulses driven by major marine carbon cycle perturbations. Nat. Geosci. 13, 745–750, https://doi.org/10.1038/s41561-020-00646-4.

Jurikova H., Abe O., Shiah F.-K., Liang M.C. (2022) New constraints on biological production and mixing processes in the South China Sea from triple isotope composition of dissolved oxygen. Biogeosciences 19, 2043–2058, https://doi.org/10.5194/bg-19-2043-2022.

Klochko K., Kaufman A. J., Wengsheng Y., Byrne R. H. and Tossell J. A. (2006) Experimental measurement of boron isotope fractionation in seawater. Earth Planet. Sci. Lett. 248, 276–285, https://doi.org/10.1016/j.epsl.2006.05.034.

Korte C. and Ullmann C.V. (2018) Permian strontium isotope stratigraphy. Geological Society, London, Special Publications 450, 105, https://doi.org/10.1144/SP450.5.

Korte C., Jasper T., Kozur H.W., Veizer J. (2005) $\delta^{18}O$ and $\delta^{13}C$ of Permian brachiopods: A record of seawater evolution and continental glaciation. Palaeogeogr. Palaeoclimatol. Palaeoecol. 224, 333–351, https://doi.org/10.1016/j.palaeo.2005.03.015.

Korte C., Jasper T., Kozur H.W., Veizer J. (2006) $^{87}Sr/^{86}Sr$ record of Permian seawater. Palaeogeogr. Palaeoclimatol. Palaeoecol. 240, 89–107, https://doi.org/10.1016/j.palaeo.2006.03.047.

Laurie J.R., Bodorkos S., Nicoll R.S., Crowley J.L., Mantle D.J., Mory A.J., Wood G.R., Backhouse J., Holmes E.K., Smith T.E., Champion D.C. (2016) Calibrating the middle and late Permian palynostratigraphy of Australia to the geologic time-scale via U-Pb zircon CA-IDTIMS dating. Aust. J. 63, 701–730, https://doi.org/10.1080/08120099.2016.1233456

Legett S.A., Rasbury E.T., Grossman E.L., Hemming N.G., and Penman D.E. (2020) The Brachiopod $\delta^{11}B$ Record Across the Carboniferous-Permian Climate Transition. Paleoceanogr. Paleoclimatol. 35, e2019PA003838, https://doi.org/10.1029/2019PA003838.

Lescaze Z. and Ford W. (2017). Paleoart: visions of the prehistoric past, 1830–1980.

Lécuyer C., Grandjean P., Reynard B., Albarède F. and Telouk P. (2002) $^{11}B/^{10}B$ analysis of geological materials by ICP-MS Plasma 54: application to the boron fractionation between brachiopod calcite and seawater. Chem. Geol. 186, 45–55, https://doi.org/10.1016/S0009-2541(01)00425-9.

Lowenstein T.K., Hardie L.A, Timofeeff M.N., Demicco R.V. (2003) Secular variation in seawater chemistry and the origin of calcium chloride basinal brines. Geology 31, 857-860, https://doi.org/10.1130/G19728R.1.

Ma X., Yana H., Fei H., Liu C., Shi G., Huang E., Wang Y., Qu X., Lian E., Dang H. (2020) A high-resolution $\delta^{18}O$ record of modern *Tridacna gigas* bivalve and its paleoenvironmental implications. Palaeogeogr. Palaeoclimatol. Palaeoecol. 554, 10980, https://doi.org/10.1016/j.palaeo.2020.109800.

McArthur J.M., Howarth R.J., Shields G.A., Zhou Y. (2020) Chapter 7 – Strontium Isotope Stratigraphy. Geologic Time Scale 2020, 211–236, https://doi.org/10.1016/B978-0-12-824360-2.00007-3.

Mii H.-S., Grossman E.L., Yancey T.E. (1999) Carboniferous isotope stratigraphies of North America: Implications for Carboniferous paleoceanography and Mississippian glaciation. Geol. Soc. Amer. Bull. 111, 960–973, ttps://doi.org/10.1130/0016-7606(1999)111<0960:CISONA>2.3.CO;2.

Montañez I.P. (2022) Current synthesis of the penultimate icehouse and its imprint on the Upper Devonian through Permian stratigraphic record. Geological Society, London, Special Publications 512, 213–245, https://doi.org/10.1144/SP512-2021-124.

Morante R. (1996) Permian and early Triassic isotopic records of carbon and strontium in Australia and a scenario of events about the Permian-Triassic boundary. Hist. Biol. 11, 289–310, https://doi.org/10.1080/10292389609380546.

Nolan L.S.P., Angiolini L., Jadoul F., Della Porta, Davies S.J., Banks V.J., Stephenson M.H., Leng M.J. (2017) Sedimentary context and palaeoecology of *Gigantoproductus* shell beds in the Mississippian Eyam Limestone Formation, Derbyshire carbonate platform, central

England. Proceedings of the Yorkshire Geological Society 61, 239–257, https://doi.org/10.1144/pygs2017-393.

Murray J.W., Roberts E., Howard E., O'Donnell M., Bantam C., Carrington E., Foy M., Paul B., Fay A. (2015) An inland sea high nitrate-low chlorophyll (HNLC) region with naturally high pCO_2. Limnol. Oceanogr. 60, 957–966, https://doi.org/10.1002/lno.10062.

Nir O., Vengosh A., Harkness J.S., Dwyer G.S., Lahav O. (2015) Direct measurement of the boron isotope fractionation factor: Reducing the uncertainty in reconstructing ocean paleo-pH. Earth Planet. Sci. Lett. 414, 1–5, https://doi.org/10.1016/j.epsl.2015.01.006.

Penman D. E., Hönisch B., Rasbury E. T., Hemming N. G. and Spero H. J. (2013) Boron, carbon, and oxygen isotopic composition of brachiopod shells: Intra-shell variability, controls, and potential as a paleo-pH recorder. Chem. Geol. 340, 32–39, https://doi.org/10.1016/j.chemgeo.2012.11.016.

Popp B.N., Anderson T.F., Sandberg P.A. (1986) Brachiopods as indicators of original isotopic compositions in some Paleozoic limestones. Geol. Soc. Amer. Bull. 97, 1262–1269, https://doi.org/10.1130/0016-7606(1986)97<1262:BAIOOI>2.0.CO;2.

Permian Timescale (2022) https://permian.stratigraphy.org/timescale, Subcommission on Permian Stratigraphy, International Commission on Stratigraphy.

Shi Y., Wang X., Fan J., Huang H., Xu H., Zhao Y., Shen S. (2021). Carboniferous-earliest Permian marine biodiversification event (CPBE) during the Late Paleozoic Ice Age. Earth Sci. Rev. 220, 103699, https://doi.org/10.1016/j.earscirev.2021.103699.

Shiino Y. and Angiolini L. (2014) Hydrodynamic advantages in the free-living spiriferinide brachiopod *Pachycyrtella omanensis*: functional insight into adaptation to high-energy flow environments. Lethaia 47, 216–228, https://doi.org/10.1111/let.12052.

Stewart J., Christopher S.J., Kucklick J.R., Bordier L., Chalk T.B., Dapigny A., Douille E., Foster G.L., Gray W.R., Greenop R., Gutjahr M., Hemsing F., Henehan M.J., Holdship P., Hsieh Y.-T., Kolevica A., Lin Y.-P., Mawbey E.M., Rae J.W.B., Robinson L.F., Shuttleworth R., You C.-F., Zhang S., Day R.D. (2020) NIST RM 8301 Boron Isotopes in Marine Carbonate (Simulated Coral and Foraminifera Solutions): Inter-laboratory $\delta^{11}B$ and Trace Element Ratio Value Assignment. Geostand Geoanal Res 45, 77–96, https://doi.org/10.1111/ggr.12363.

Šimůnek Z., & Martínek K. (2009). A study of Late Carboniferous and Early Permian plant assemblages from the Boskovice Basin, Czech Republic. Review of Palaeobotany and Palynology 155, 275–307, https://doi.org/10.1016/j.revpalbo.2008.05.006.

Trudgill M., Nuber S., Block H., Crumpton-Banks J., Jurikova H., Littley E., Shankle M., Xu C., Steele R.C.J., Rae J.W.B. (2024) A simple, low-blank batch purification method for high-precision boron isotope analysis. Geochem. Geophys. Geosyst. 25, e2023GC011350, https://doi.org/10.1029/2023GC011350.

Viaretti M., Heward A.P., Gementi A., and Angiolini L. (2022) Upper Cisularian-Lower Guadalupian brachiopods from the Qarari Unit, Batain plain, northeast Oman: systematics, palaeoecology and correlation. Riv. Ita. Paleontol. Strat. 128, 643–694, https://doi.org/10.54103/2039-4942/17732.

Veizer J. and Compston W. (1974) $^{87}Sr/^{86}Sr$ composition of seawater during the Phanerozoic. Geochim. Cosmochim. Acta 38, 1461–1484, https://doi.org/10.1016/0016-7037(74)90099-4.

Veizer J., Ala D., Azmy K., Bruckschen P., Buhl D., Bruhn F., Carden G.A.F., Diener A., Ebneth S., Goddéris Y., Jasper T., Korte C., Pawellek F., Podlaha O.G., Strauss H. (1999) $^{87}Sr/^{86}Sr$, $\delta^{13}C$, $\delta^{18}O$ evolution of Phanerozoic seawater. Chem. Geol. 161, 59–58, https://doi.org/10.1016/S0009-2541(99)00081-9.

Viaretti M., Heward A.P., Gementi A., Angiolini L. (2022) Upper Cisuralian-lower Guadalupian brachiopods from the Qarari Unit, Batain plain, northeast Oman: systematics,

palaeoecology and correlation. Riv. Ital. Paleontol. Stratigr. 128, 643–694, https://doi.org/10.54103/2039-4942/17732.
Vollstaedt H., Eisenhauer A., Wallmann K., Böhm, F., Fietzke J., Liebetrau V., Krabbenföft A., Farkaš J., Tomašových A., Raddatz J., Viezer J. (2014) The Phanerozoic $\delta^{88/86}$Sr record of seawater: New constraints on past changes in oceanic carbonate fluxes. Geochim. Cosmochim. Acta 128, 249–265, https://doi.org/10.1016/j.gca.2013.10.006.
Wang W., Garbelli C., Zheng Q., Chen J., Liu X., Wang W., Shen S. (2018) Permian ^{87}Sr/^{86}Sr chemostratigraphy from carbonate sequences in South China. Palaeogeogr. Palaeoclimatol. Palaeoecol. 500, 84–94, https://doi.org/10.1016/j.palaeo.2018.03.035.
Wang W., Garbelli C., Zhang F., Zheng Q., Zhang Y., Yuan D., Shi Y.,Chen B., Shen S. (2020) A high-resolution Middle to Late Permian paleotemperature curve reconstructed using oxygen isotopes of well-preserved brachiopod shells. Earth Planet. Sci Lett. 540, 116245, https://doi.org/10.1016/j.epsl.2020.116245.
Wang W., Katchinoff J.A.R., Garbelli C., Immenhauser A., Zheng Q., Zhang Y., Yuan D., Shi Y., Wang J., Planavsky N., Shen S. (2021) Revisiting the Permian seawater ^{87}Sr/^{86}Sr record: New perspectives from brachiopod proxy data and stochastic oceanic box models. Earth Sci. Rev. 218, 103679, https://doi.org/10.1016/j.earscirev.2021.103679
Whiteford R., Heaton T.J., Henehan M.J., Anagnostou E., Jurikova H., Foster G.L., Rae J.W.B. (2023) Reconstruction of Cenozoic $\delta^{11}B_{sw}$ Using A Gaussian Process. Paleoceanogr. Paleoclimatol. In review, https://doi.org/10.22541/essoar.169603519.93470287/v1.
Wierzbowski H., Anczkiewicz R., Pawlak J., Rogov M.A., Kuznetsov A.B. (2017) Revised Middle-Upper Jurassic strontium isotope stratigraphy. Chem Geol. 466, 239–255, https://doi.org/10.1016/j.chemgeo.2017.06.015.
Ye F., Crippa G., Angiolini L., Brand U., Capitani G., Cusack M., Garbelli C, Griesshaber E., Harper E.M., Schmahl W.W. (2018) Mapping of recent brachiopod microstructure: A tool for environmental studies. J. Struct. Biol. 201, 221–236, https://doi.org/10.1016/j.jsb.2017.11.011.
Wu H.C., Dissard D., Douville E., Blamart D., Bordier L., Tribollet A., Le Cornec F., Pons-Branchu E., Dapoigny A., Lazareth C.E. (2018) Surface ocean pH variations since 1689 CE and recent ocean acidification in the tropical South Pacific. Nat. Comm. 9, 2543, https://doi.org/10.1038/s41467-018-04922-1.
Xu C., Jurikova H., Nuber S., Steele R.C.J., Trudgill M., Barker S., Lear C.H., Burke A., Rae J.W.B (2024) A rapid, simple, and low-blank pumped ion-exchange column chromatography technique for boron purification from carbonate and seawater matrices. Geochem. Geophys. Geosyst. 25, e2023GC011228, https://doi.org/10.1029/2023GC011228.
Zhai W. (2019) Exploring seasonal acidification in the Yellow Sea. Sci. China Earth Sci. 61, 647–658, https://doi.org/10.1007/s11430-017-9151-4.
